# Born’s Rule from Contextual Relative-Entropy Minimization

**DOI:** 10.3390/e27090898

**Published:** 2025-08-25

**Authors:** Arash Zaghi

**Affiliations:** College of Engineering, University of Connecticut, Unit 3037, 261 Glenbrook Rd., Storrs, CT 06269, USA; arash.esmaili_zaghi@uconn.edu

**Keywords:** Petz’s projection theorem, umegaki relative entropy, sheaf-theoretic contextuality, born rule, relational quantum mechanics (RQM), integrated information, measurement contexts (MASAs)

## Abstract

We give a variational characterization of the Born rule. For each measurement context, we project a quantum state *ρ* onto the corresponding abelian algebra by minimizing Umegaki relative entropy; Petz’s Pythagorean identity makes the dephased state the unique local minimizer, so the Born weights $p_{C}\left(i\right)=\mathrm{Tr}\left(\rho P_{i}\right)$ arise as a consequence, not an assumption. Globally, we measure contextuality by the minimum classical Kullback–Leibler distance from the bundle $\left\{p_{C}\left(\rho \right)\right\}$ to the noncontextual polytope, yielding a convex objective Φ(ρ). Thus, Φ(ρ)=0 exactly when a sheaf-theoretic global section exists (noncontextuality), and Φ(ρ)>0 otherwise; the closest noncontextual model is the classical *I*-projection of the Born bundle. Assuming finite dimension, full-rank states, and rank-1 projective contexts, the construction is unique and non-circular; it extends to degenerate PVMs and POVMs (via Naimark dilation) without change to the statements. Conceptually, the work unifies information-geometric projection, the presheaf view of contextuality, and categorical classical structure into a single optimization principle. Compared with Gleason-type, decision-theoretic, or envariance approaches, our scope is narrower but more explicit about contextuality and the relational, context-dependent status of quantum probabilities.

## 1. Introduction

Modern quantum theory still rests on an empirical prescription—the Born rule—that converts the formal wave function into concrete outcome frequencies. Nearly a century after Born’s original proposal, the rule remains the last standing axiom that resists unanimous reduction to deeper principles [1,2]. In this paper, we attempt to close that gap by showing that the Born probabilities arise uniquely from a single variational requirement: minimize the information-geometric distance to the non-contextual polytope across all measurement contexts. This derivation weaves together three previously disparate strands: (i) Umegaki--Petz relative entropy projections inside each maximal abelian sub-algebra, (ii) the sheaf-cohomological obstruction that defines contextuality, and (iii) a relational, observer-relative ontology. The result elevates the Born rule from an axiom to the least-disturbance bridge between incompatible classical standpoints, thereby reconciling quantum probability with the demands of contextuality, relativity of states, and categorical naturality.

Max Born’s 1926 [1] insight that ${\left|\psi \right|}^{2}$ yields statistical weights created an operational rule that Dirac soon canonized in the Principles [3]. Ever since, theorists have sought a derivation from first principles. Gleason’s measure-theoretic theorem secures the trace form on Hilbert spaces of dimension ≥ 3, but only by postulating a non-contextual frame function that is itself stronger than what any single measurement requires [4]. The Kochen–Specker theorem later showed that such a globally non-contextual frame cannot exist at all [5,6]. Alternative programmes invoke special physical assumptions: Zurek’s envariance symmetry recovers equal-amplitude cases yet still needs continuity to reach arbitrary moduli [7,8,9]; the Deutsch-Wallace decision-theoretic route derives quantum credences from rational preferences inside the Everett picture [10,11,12]; Hartle’s frequency-operator spectra tie probabilities to infinite repetition limits [13]; Bayesian reconstructions exploit exchangeability in quantum de Finetti theorems [14]; Busch’s POVM-Gleason generalisation closes the qubit loophole by enlarging the effect space [15]; and operational reconstructions à la Hardy and Chiribella–D’Ariano–Perinotti start from abstract information-processing axioms [16,17]. Each story illuminates part of the landscape, yet all smuggle in extra structure—continuity, rationality, purification, or non-contextuality—whose physical inevitability remains debated.

The modern moral is that quantum probability is intrinsically contextual [6,18]. Sheaf theory makes this precise by treating a “measurement scenario” as a cover of contexts and identifying contextuality with the obstruction to a global section [19]. Cohomological refinements classify the obstruction and reveal hierarchies of contextual strength [20,21,22], while information-theoretic measures such as the relative-entropy of contextuality supply quantitative monotones [23]. Our work adopts this viewpoint wholesale: the non-contextual polytope is the reference body, and “distance” from it is the resource cost of contextuality.

Relative entropy furnishes our notion of statistical deviation. Umegaki introduced the quantum version in 1962 [24]; it is jointly convex, strictly convex in its first argument, and monotone under CPTP maps (data processing) [25]. Petz showed that conditional expectation onto a von Neumann subalgebra satisfies a Pythagorean identity, which implies that dephasing onto a context (MASA) uniquely minimizes S(ρ∥σ) over classical states in that context [26]. On the classical side, Csiszár characterized KL minimizers as *I*-projections under linear expectation constraints [27]. We use exactly these two facts: (i) per-context dephasing as the unique quantum projection, and (ii) exponential-family solutions for linear constraints.

Our construction is relational in Rovelli’s sense: states are attributes of interactions, not intrinsic properties [28,29,30]. In categorical quantum mechanics, each context corresponds to a special commutative dagger Frobenius algebra (SCFA) on *H*; its copy/delete maps (δ,ε) implement classical data, and the associated decoherence (dephasing) idempotent realizes the passage from quantum to classical within that context [31,32]. In the completely positive maps (CPM) semantics, probabilities are scalars obtained by composing states with effects; for SCFAs in FHilb these scalars are precisely the Born weights $\mathrm{Tr}(\rho P_{i})$. Our variational principle respects this naturality: the local information projection is exactly the SCFA-induced dephasing, and the global step chooses the least-informative joint consistent with the noncontextual constraints. Therefore, the rule selected by the category is the same one singled out by the optimization—Born’s rule—in harmony with relational/process-theoretic accounts [28].

Building on the preliminaries, we proceed in four steps. (1) We quantify contextuality by the contextual divergence Φ(ρ): the minimum (weighted) classical KL distance from the empirical bundle $p_{M}\left(\rho \right)={\left\{p_{C}\left(\rho \right)\right\}}_{C\in M}$ to the noncontextual set NC. (2) For each context *C* (a MASA; see the glossary), Petz’s Pythagorean identity implies that the information projection of ρ onto the classical face S(C) is the dephasing $E_{C}\left(\rho \right)$, which fixes the Born weights $p_{C}\left(i\right)=\mathrm{Tr}\left(\rho P_{i}\right)$ without assuming them. (3) Any globally consistent assignment that is locally entropy-optimal in every context must therefore use these Born weights; the global problem reduces to the classical *I*-projection of the Born bundle onto NC. When the cover is contextual, the minimizer deviates minimally from Born in order to satisfy global consistency, and Φ(ρ)>0. (4) We interpret the resulting probabilities as the least-informative classical summaries compatible with the relational structure of the cover, linking the sheaf-theoretic obstruction to an explicit optimization principle.

Appendix A extends the analysis to degenerate projection-valued measures (PVMs) and general positive operator-valued measures (POVMs) via Naimark dilation, providing the corresponding exponential-family (quantum Jeffrey) updates and showing that all statements are dilation-stable (Lüders appears only in the projective case). Appendix B gathers the convex-optimization machinery: the existence of a minimizer, a full Karush-Kuhn-Tucker (KKT) that handle zero-probability coordinates, and the uniqueness of the optimal marginals. Under informational completeness and noncontextuality, these marginals identify the generating state ρ uniquely.

In short, the Born trace rule is not postulated but forced by a two-stage variational principle: local least-disturbance (dephasing) plus a global *I*-projection onto NC. Contextuality is quantified by Φ(ρ)—the minimal information cost of fitting a single classical narrative—and the relational stance is built into the very form of the optimization. No other assignment simultaneously minimizes information loss and remains as classical as the contextual fabric of the scenario allows.

For readability we define all acronyms at first use and provide a consolidated ‘Abbreviations and Symbols’ table before the appendices.

## 2. Mathematical Preliminaries

### 2.1. Hilbert Space, Contexts, and Empirical Models

We work with a finite-dimensional Hilbert space H and fix once-and-for-all a cover M of measurement contexts, with each context *C* being a maximal abelian subalgebra of B(H), i.e., a commuting set of projectors $\left\{P_{i}^{C}\right\}$ summing to the identity. Crucially, M is chosen independently of any state, so we do not “tailor” contexts to ρ.

For a state ρ, each context *C* yields an empirical distribution $p_{C}\left(i;\rho \right)\;=\;F\left(\rho ,\;P_{i}^{C},\;C\right),$ assigning to outcome $P_{i}^{C}$ the probability $p_{C}\left(i;\rho \right)$. In orthodox quantum theory $F\left(\rho ,P_{i}^{C},C\right)=\mathrm{Tr}\left(\rho \;P_{i}^{C}\right)$, but here we do not assume the Born rule. Rather, we collect all these $C\mapsto p_{C}\left(\cdot ;\rho \right)$ into a presheaf of distributions over M: whenever two contexts $C⊃C^{\prime }$ overlap, the full distribution $p_{C}$ restricts to the marginal on $C^{\prime }$. Our goal is to show—via a simple variational principle—that the only way these context-wise shadows can consistently arise from a single density operator is if *F* collapses to the familiar trace form.

A state ρ is noncontextual for the cover M if there exists a single joint distribution *g* over all outcomes X=⋃M whose marginal on each context *C* agrees with $p_{C}\left(\cdot ;\rho \right)$ [33]. If no such *g* exists, the empirical model is contextual, reflecting the Kochen–Specker obstruction to a hidden-variable assignment consistent across all *C* [6]. In sheaf-cohomological language, contextuality is witnessed by a nontrivial class in the first Čech cohomology ${\overset{ˇ}{H}}^{1}\left(M,F\right)$: the local $p_{C}$ form a 1-cocycle that fails to glue into a global section [19,21]. A vanishing class is therefore both necessary and sufficient for a global classical model of the data.

Noncontextual behaviors form a convex polytope $\mathrm{NC}\subset \prod_{C\in M}\Delta_{C}$; namely, all families $\left\{g_{C}\right\}$ admitting a global joint *g* on *X* with marginals $g_{C}$ [33]. Equivalently, NC is the convex hull of deterministic value assignments. In d≥3, most quantum empirical models lie outside NC, while for qubits one needs a Kochen–Specker configuration to see contextuality [6]. Rather than seek an exact (and generally impossible) global section, we will measure contextuality by the distance of $\left\{p_{C}\right\}$ from NC.

### 2.2. Umegaki Relative Entropy as Divergence Measure

To gauge how far a quantum empirical model $\left\{p_{C}\right\}$ lies outside the noncontextual polytope NC, we employ the Umegaki–Petz relative entropy [24]. For two states ρ,σ on H,

(1) $$S\left(\rho ∥\sigma \right)=\mathrm{Tr}\left[\rho \;(\ln\rho -\ln\sigma )\right],$$

defined whenever supp(ρ)⊆supp(σ) (and +∞ otherwise). As the quantum analogue of classical KL, S(ρ∥σ)≥0 with equality iff ρ=σ on the stated support domain. It is strictly convex in its first argument ρ↦S(ρ∥σ) and jointly convex in (ρ,σ); it obeys the data-processing inequality for every CPTP (for abbreviations refer to the end of this document before the appendices) map Λ,

$$S\left(\Lambda (\rho )\;∥\;\Lambda (\sigma )\right)\;\leq \;S\left(\rho ∥\sigma \right),$$

so coarse-grainings never increase the divergence. It is unitarily invariant under simultaneous conjugation, $S\left(U\rho U^{†}∥U\sigma U^{†}\right)=S\left(\rho ∥\sigma \right)$, but in general does not depend only on the spectra of ρ and σ unless they commute [34]. Though not a metric, its lower semicontinuity, convexity, and monotonicity under coarse-graining make it the canonical divergence for projecting quantum states onto commutative (classical) models; in those settings the objective reduces to a classical KL and, under full-support conditions, yields a unique minimizer.

Two features of the Umegaki–Petz relative entropy make it ideal for our variational framework. First, S(τ∥ρ) is strictly convex in its first argument and jointly convex in (τ,ρ). Hence (a) in the POVM/overlap problems where we minimize S(τ∥ρ) over τ subject to linear expectation constraints, the optimizer $\tau^{★}$ is unique; and (b) in the per-context projection $\min_{\sigma \in S(C)}S\left(\rho ∥\sigma \right)$, Petz’s identity reduces the objective to a classical KL $D_{\mathrm{KL}}\;\left(p_{C}\left(\rho \right)∥q\right)$ (with *q* the diagonal of σ), which is strictly convex in *q*, yielding the unique solution $E_{C}\left(\rho \right)$ whenever $p_{C}\left(\rho \right)$ has full support [25,26]. See Appendix A for the POVM/degenerate case via Naimark dilation and exponential-family updates. Second, we use Petz’s Pythagorean identity for the conditional expectation (dephasing) onto *C*,

$$S\left(\rho ∥\sigma \right)=S\left(\rho ∥E_{C}\left(\rho \right)\right)+S\left(E_{C}\left(\rho \right)∥\sigma \right)\;\left(\sigma \in S\left(C\right)\right),$$

which splits the divergence into a context-dependent term plus a purely classical term. This decouples the optimization across contexts and then lets us glue the optimal marginals consistently.

### 2.3. Sheaf-Theoretic View of Noncontextuality and Divergence

In a fixed measurement scenario (X,M), let *X* be a set of rank-1 projectors and M a cover by contexts C⊂X (each a maximal commuting set), with outcomes $O_{x}=\left\{0,1\right\}$. A state ρ defines an empirical presheaf $C\mapsto p_{C}\left(\cdot ;\rho \right)$ whose marginals agree on every overlap $C\cap C^{\prime }$. Categorically, $\left\{p_{C}\right\}$ is a Čech 1-cocycle, and ρ is noncontextual exactly if this cocycle is a coboundary—i.e., there is a global section *g* with $p_{C}{=g|}_{C}$ for all *C*. If no such *g* exists, the resulting nonzero class in ${\overset{ˇ}{H}}^{1}\left(M,F\right)$ certifies contextuality [21].

We introduce a quantitative measure of contextuality using the divergence defined above. Recall that the Umegaki relative entropy S(ρ∥σ) is defined when supp(ρ)⊆supp(σ) (and +∞ otherwise); it is strictly convex in its first argument and jointly convex in (ρ,σ), obeys data processing S(Λ(ρ)∥Λ(σ))≤S(ρ∥σ) for all CPTP Λ, and is unitarily invariant under simultaneous conjugation, though not determined solely by the spectra unless ρ and σ commute. Intuitively, we ask: “How much must one alter ρ’s empirical model to make it noncontextual?” This leads to the contextual divergence Φ(ρ), defined as the minimal information divergence between the quantum model and any noncontextual model in NC. Formally, let $p_{M}\left(\rho \right)={\left\{p_{C}\left(\cdot ;\rho \right)\right\}}_{C\in M}$ denote the full bundle of contextual distributions for ρ. We define:

(2) $$\Phi \left(\rho \right)\;=\;\min_{\;g\in \mathrm{NC}}\;S\left(p_{M}\left(\rho \right)\;∥\;g\right)\;.$$

where here $S\left(p_{M}\left(\rho \right)\;∥\;g\right)$ denotes the aggregate classical divergence, for example,

$$\sum_{C\in M}\mu_{C}\;D_{\mathrm{KL}}\left(p_{C}\left(\rho \right)\;∥\;g_{C}\right),$$

tagging each outcome by its context to avoid double counting. We assume NC≠⌀ (otherwise interpret Φ(ρ)=+∞ or use an ε-noisy relaxation).

Our derivation will enforce the principle that Φ(ρ) be minimized. In other words, we seek an assignment of probabilities to measurement outcomes that makes a given state ρ as nearly noncontextual as possible. Subject to the usual constraints of quantum probabilities, such as normalization, positivity, and the functional relations imposed by projectors, we will find that this variational principle singles out a unique assignment—one that turns out to coincide with the Born rule. Crucially, this conclusion will emerge without ever assuming the Born rule in advance as we have treated $p_{C}\left(i;\rho \right)$ abstractly so far. Rather, the trace-form $p_{C}\left(i\right)=\mathrm{Tr}\left(\rho P_{i}\right)$ will appear as a consequence of minimizing information divergence under the structural constraints of locality and global consistency.

### 2.4. Categorical Framework and Classical Structures

Before the analytical proof, we recast the problem in categorical quantum mechanics [35], which makes explicit the structural ingredients—quantum states, measurement contexts, and probabilistic outcomes. We model our system as an object *A* in a dagger-compact symmetric monoidal category C, with processes as morphisms. We assume C supports abstract states, effects, and—for each measurement context *C*—a commutative †-Frobenius algebra on *A* that encodes the classical copy-and-delete structure for that basis.

#### 2.4.1. Commutative Frobenius Algebra

A special commutative Frobenius algebra on an object *A* consists of

m:A⊗A→A,u:I→A,δ:A→A⊗A,ϵ:A→I,

satisfying the usual Frobenius and unit laws. Intuitively, δ duplicates and ϵ discards classical data in *A*. Commutativity means m∘τ=m (inputs unordered), and the “special’’ condition $m\circ \delta ={\mathrm{id}}_{A}$ ensures copying then merging returns the original.

In FHilb, each orthonormal basis {|i〉} of *H* yields such an algebra. The basis vectors arise as the unique comonoid homomorphisms (classical points)

$$\delta_{i}:I\to A,\;\delta_{i}\left(1\right)=|i\rangle ,$$

and their adjoints $\delta_{i}^{†}:A\to I$ are the corresponding effects. Concretely, on basis vectors

δ(|i〉)=|i〉⊗|i〉,ϵ(|i〉)=1,

extended linearly, while $m\left(|i\rangle ⊗|j\rangle \right)=\delta_{ij}\;|i\rangle ,$ one convenient (unnormalized) choice of unit is $u\left(1\right)=\sum_{i}|i\rangle \;.$

#### 2.4.2. States and Effects as Morphisms

A pure state is the morphism |ψ〉:I⟶A, sending 1 to |ψ〉. In CPM one represents it instead as the density-operator morphism

ρ=|ψ〉〈ψ|:I⟶A.

Each classical point $\delta_{i}$ induces a projector $P_{i}\;=\;\delta_{i}\circ \delta_{i}^{†}\;=\;|i\rangle \langle i|,$ and the Born probability is obtained by composing with ρ:

$$I\overset{\;\rho \;}{\to }A\overset{\;P_{i}\;}{\to }I\;=\;\mathrm{Tr}\left(P_{i}\;\rho \right)\;=\;{\left|\langle i|\psi \rangle \right|}^{2}.$$

Equivalently, one may insert the bra morphism explicitly:

$$I\overset{\;|\psi \rangle \;}{\to }A\overset{\;P_{i}\;}{\to }A\overset{\;\langle \psi |\;}{\to }I\;=\;\langle \psi |\;P_{i}\;|\psi \rangle \;=\;{\left|\langle i|\psi \rangle \right|}^{2}.$$

#### 2.4.3. Unified Effect

Define for context *C* the effect

$${!}_{i}^{C}\;=\;\delta_{i}^{†}\circ P_{i}^{C}\;:\;A⟶I.$$

Then for a state ρ (pure or mixed),

$$\mathrm{Pr}\left(i|\rho ,C\right)=\;{!}_{i}^{C}\circ \rho :I⟶I,$$

which in FHilb evaluates to $\langle \psi |P_{i}^{C}|\psi \rangle =\mathrm{Tr}\left(P_{i}^{C}\;\rho \right)$, recovering the Born rule.

Axiomatic scope: In this work we assume from the outset that our ambient category is dagger-compact, or equivalently that each †-SCFA carries a faithful Frobenius trace. All subsequent KL-minimisation and Born-rule emergence rest on that dagger/trace structure; no further inner-product or Gleason-type postulate is invoked.

Crucially, one can show from the Frobenius-algebra axioms (copying, deleting, and monoidal composition) that this is the only way to produce a well-defined real scalar from a state–outcome pair. Hence, once a classical context structure is assumed and probabilities are required to be scalar morphisms in a monoidal category, the usual Born rule is forced: compatibility with classical structures and functoriality uniquely picks out the Hilbert-space trace as the probability assignment.

#### 2.4.4. Categorical Consistency Check

In FHilb, each context carries a commutative †-Frobenius algebra that supplies copy/delete for classical data. Composing a state with the effect and the counit yields a scalar that, in FHilb, evaluates to $\mathrm{Tr}(\rho P_{i})$. Thus, the variationally selected weights coincide with the scalar produced by the standard categorical interface. We use this as a consistency check rather than an independent derivation.

In summary, the categorical formulation assures us that nothing mysterious is hiding in our choice of measurement contexts: each context *C* supplies a classical interface (copy/delete operations) through which quantum states produce scalar outcomes. The Born rule appears as the inevitable scalar morphism arising from composing a state with a context’s effect and the counit (discard) map. This provides a high-level consistency check for our approach: any variational or information-theoretic argument we make in the Hilbert-space formalism will align with the fundamental categorical structure that already encapsulates the Born rule. In particular, it means that if our optimization principle selects a unique candidate for $p_{C}\left(i;\rho \right)$, that candidate must correspond to $\mathrm{Tr}(\rho P_{i}^{C})$ in the concrete model—otherwise it would contradict the established classical interface of FHilb. With this assurance, we now proceed to the core of the argument: identifying the optimal local classical approximations and understanding how (and whether) they can be “glued” into a global noncontextual model.

## 3. Quantifying Contextuality Locally and Globally

This section turns contextuality from a logical obstruction into a quantitative optimization problem and extracts the Born weights from a local variational principle. *Locally* (Section 3.1), we show that for each context *C* the Umegaki–Petz projection sends ρ to its dephasing $E_{C}\left(\rho \right)$, thereby fixing the Born probabilities $p_{C}\left(i\right)=\mathrm{Tr}\left(\rho P_{i}\right)$ without assuming them. *Globally* (Section 3.2 and Section 3.3), we compare the Born bundle ${\left\{p_{C}\left(\rho \right)\right\}}_{C\in M}$ to the noncontextual set NC and define the contextual divergence Φ(ρ), which vanishes exactly on noncontextual models and otherwise quantifies the minimal information cost of enforcing a single classical narrative. We then prove a two-stage theorem: the only locally entropy-optimal assignments are the Born weights, and the best global fit is the classical *I*-projection of the Born bundle onto NC. Existence/uniqueness (and handling zero-probability entries) are deferred to Appendix B; POVM/degenerate contexts follow by Naimark dilation in Appendix A.

### 3.1. Optimal Classical Approximations in a Single Context

#### 3.1.1. Setup and Notation

Fix a context C∈M given by a projective measurement $\left\{P_{i}:i\in I_{C}\right\}$ on a finite–dimensional Hilbert space H, with $P_{i}P_{j}=\delta_{ij}P_{i}$ and $\sum_{i}P_{i}=1$. Let $r_{i}:=\mathrm{rank}\left(P_{i}\right)$. We write $\pi_{i}:=P_{i}/r_{i}$ (the maximally mixed state on the *i*th outcome subspace.) The classical states on context *C* form the convex set

$$S\left(C\right)\;:=\;\{\sigma \in D\left(H\right)\;:\;\sigma =\sum_{i\in I_{C}}q_{i}\;\pi_{i}\;\;\mathrm{for}\;\mathrm{some}\;\mathrm{probability}\;\mathrm{vector}\;q={\left(q_{i}\right)}_{i}\}.$$

For rank–1 contexts ($r_{i}=1$ for all *i*), this reduces to $S\left(C\right)=\left\{\sigma =\sum_{i}q_{i}P_{i}\right\}$.

#### 3.1.2. Conditional Expectation (Dephasing) onto *C*

Let $E_{C}:B\left(H\right)\to \mathrm{span}\left\{P_{i}\right\}$ be the trace-preserving conditional expectation onto the abelian von Neumann subalgebra generated by $\left\{P_{i}\right\}$ (the “dephasing” onto *C*). Concretely,

(3) $$E_{C}\left(X\right)\;=\;\sum_{i\in I_{C}}\frac{\mathrm{Tr}(P_{i}X)}{r_{i}}\;P_{i}\;=\;\sum_{i\in I_{C}}\mathrm{Tr}\left(X\;\pi_{i}\right)\;P_{i}\;\left(X\in B\left(H\right)\right).$$

This map satisfies $E_{C}\left(\sigma \right)=\sigma$ for all σ∈S(C) and

(4) $$\mathrm{Tr}\left(E_{C}\left(X\right)\;A\right)\;=\;\mathrm{Tr}\left(X\;A\right)\;\mathrm{for}\;\mathrm{all}\;A\in \mathrm{span}\left\{P_{i}\right\}.$$

#### 3.1.3. The Pythagorean Identity (Petz)

Write the Umegaki relative entropy

$$S\left(\rho ∥\sigma \right)\;:=\;\mathrm{Tr}\left[\rho (\log\rho -\log\sigma )\right]$$

whenever supp(ρ)⊆supp(σ) (and +∞ otherwise). Petz’s decomposition theorem gives the Pythagorean identity for the conditional expectation $E_{C}$ (see, e.g., [26]):

(5) $$S\left(\rho ∥\sigma \right)\;=\;S\left(\rho ∥E_{C}\left(\rho \right)\right)\;+\;S\left(E_{C}\left(\rho \right)∥\sigma \right)\;\mathrm{for}\;\mathrm{every}\;\sigma \in S\left(C\right).$$

The first term is independent of σ.

#### 3.1.4. Consequences of Equation (5)

Equation (5) immediately yields that the unique minimizer of S(ρ∥σ) over σ∈S(C) is $E_{C}\left(\rho \right)$, provided the usual interior (full-support) condition holds in this context (stated below). Moreover, since $E_{C}\left(\rho \right)\in S\left(C\right)$ and every σ∈S(C) commutes with $E_{C}\left(\rho \right)$, the second term is classical:

(6) $$S\left(E_{C}\left(\rho \right)∥\sigma \right)\;=\;D_{\mathrm{KL}}\left(p∥q\right),$$

where

$$p_{i}\;:=\;\mathrm{Tr}\left(E_{C}\left(\rho \right)P_{i}\right)\;=\;\mathrm{Tr}\left(\rho P_{i}\right),\;q_{i}\;:=\;\mathrm{Tr}\left(\sigma P_{i}\right),$$

and $D_{\mathrm{KL}}\left(p∥q\right)=\sum_{i}p_{i}\left(\log p_{i}-\log q_{i}\right)$ is the classical Kullback–Leibler divergence. The equality $p_{i}=\mathrm{Tr}\left(\rho P_{i}\right)$ follows from Equation (4) with $A=P_{i}$; it is thus a consequence of the variational setup rather than an assumption.

**Proposition** **1** (Optimal classical state in a context)**.** *For any density operator ρ and any context C with projectors $\left\{P_{i}\right\}$,*

$$\arg\min_{\sigma \in S(C)}S\left(\rho ∥\sigma \right)\;=\;E_{C}\left(\rho \right)\;=\;\sum_{i\in I_{C}}\frac{\mathrm{Tr}(\rho P_{i})}{r_{i}}\;P_{i}\;=\;\sum_{i\in I_{C}}p_{C}\left(i;\rho \right)\;\pi_{i},$$

*where $p_{C}\left(i;\rho \right):=\mathrm{Tr}\left(\rho P_{i}\right)$. In the rank–1 case this reads $E_{C}\left(\rho \right)=\sum_{i}\mathrm{Tr}\left(\rho P_{i}\right)\;P_{i}$.*

**Proof (one line via Equation** **(5)).** By Equation (5), $S\left(\rho ∥\sigma \right)=S\left(\rho ∥E_{C}\left(\rho \right)\right)+S\left(E_{C}\left(\rho \right)∥\sigma \right)$ for all σ∈S(C). The second term is minimized if and only if $\sigma =E_{C}\left(\rho \right)$. Uniqueness holds whenever $p_{i}>0$ for all *i* (strict convexity of $D_{\mathrm{KL}}$ on the simplex interior). The displayed formula for $E_{C}\left(\rho \right)$ follows from Equation (3). □

#### 3.1.5. Interpretation

$E_{C}\left(\rho \right)$ is the information projection of ρ onto the classical face S(C): it preserves exactly the measurement statistics of ρ in context *C* and discards all phases (coherences) that *C* cannot detect. In particular, $\mathrm{Tr}\left(E_{C}\left(\rho \right)P_{i}\right)\;=\;\mathrm{Tr}\left(\rho P_{i}\right)$ for each *i*, so the usual trace formula for outcome weights appears naturally at the minimizer.

#### 3.1.6. Remarks on Degeneracy, Support, and Uniqueness

- Degenerate outcomes. When $r_{i}>1$, every σ∈S(C) is block-constant, $\sigma =\sum_{i}q_{i}\pi_{i}$, and $E_{C}\left(\rho \right)=\sum_{i}p_{i}\pi_{i}$ with $p_{i}=\mathrm{Tr}\left(\rho P_{i}\right)$. In this case Equation (6) holds verbatim and the proof does not change.
- Support. If some $p_{i}=0$ then the minimizer need not be unique on the face $\left\{q_{i}\;=\;0\;\mathrm{whenever}\;p_{i}=0\right\}$, but $E_{C}\left(\rho \right)$ is always a minimizer. If $p_{i}>0$ for all *i* (equivalently, $E_{C}\left(\rho \right)$ has full support in S(C)) then the minimizer is unique.
- No “chain-rule” needed. The argument uses only the Pythagorean identity Equation (5) for the conditional expectation $E_{C}$; it does not assume Born weights in advance and it avoids ill-defined terms such as $S({\tilde{\rho }}_{i}∥P_{i})$.

### 3.2. Consistency on Overlaps and the Contextual Obstruction

#### 3.2.1. Local Compatibility from Dephasing

Fix a measurement cover M of contexts C⊆X and, for each C∈M, write the optimal classical approximation (information projection) as

$$E_{C}\left(\rho \right)=\sum_{i\in I_{C}}p_{C}\left(i;\rho \right)\;\pi_{i},\;p_{C}\left(i;\rho \right):=\mathrm{Tr}\left(\rho P_{i}\right),\;\;\pi_{i}:=\frac{P_{i}}{\mathrm{rank}P_{i}},$$

as established in the previous subsection. If $C,C^{\prime }\in M$ share an outcome projector $P\;\in \;C\cap \;C^{\prime }$, then

$$\mathrm{Tr}\left(E_{C}\left(\rho \right)\;P\right)=\mathrm{Tr}\left(\rho P\right)=\mathrm{Tr}\left(E_{C^{\prime }}\left(\rho \right)\;P\right),$$

and, more generally, the marginals of $E_{C}\left(\rho \right)$ and $E_{C^{\prime }}\left(\rho \right)$ agree on the overlap $C\cap C^{\prime }$. Thus, the family of classical distributions ${\left\{p_{C}\left(\rho \right)\right\}}_{C\in M}$ obtained from the dephasings forms a compatible 0-cochain on the presheaf of outcome distributions: restrictions to overlaps coincide by construction.

#### 3.2.2. Global Sections and Noncontextual Models

Let D(U) denote the simplex of probability distributions on the outcomes of a measurement set U⊆X, with restriction maps given by marginalization. A global section is a distribution g∈D(X) whose marginals $g_{C}$ match the empirical data in every context:

$$g_{C}={\mathrm{res}}_{X\to C}\left(g\right)\;\;\;\mathrm{for}\;\mathrm{all}\;C\in M,\;\mathrm{and}\;g_{C}=p_{C}\left(\rho \right).$$

Following the sheaf-theoretic formulation, the empirical model $\left\{p_{C}\left(\rho \right)\right\}$ is noncontextual iff a global section exists; failure of existence is exactly contextuality. In our setting, this means that although every pair (indeed every finite family) of contexts agrees on its overlaps, there may be no single joint *g* on *X* that glues all contexts simultaneously.

#### 3.2.3. Cohomological Witness (Čech Obstruction)

There is a canonical way to assign to a compatible 0-cochain a cohomology class $\left[z\right]\in {\overset{ˇ}{H}}^{1}\left(M,F\right)$ (for a suitable abelian coefficient presheaf F derived from the support). If [z]≠0 then no global section exists, certifying contextuality. This obstruction is sufficient but not necessary in full generality; vanishing of [z] does not guarantee noncontextuality in every scenario. We use it as a robust witness rather than a complete characterization.

#### 3.2.4. Quantifying the Obstruction by an Optimal Global Glue

Denote by NC the noncontextual polytope: the set of all joint distributions g∈D(X) whose marginals $\left\{g_{C}\right\}$ are obtained from convex mixtures of deterministic global assignments (equivalently, the convex hull of {0,1}-valued global sections consistent with the functional relations). We quantify the failure to glue by the convex program

(7) $$\Phi \left(\rho \right)\;:=\;\min_{\;g\in \mathrm{NC}}\;\sum_{C\in M}\mu_{C}\;D_{\mathrm{KL}}\left(p_{C}\left(\rho \right)\;∥\;g_{C}\right),\;\mu_{C}>0,\;\sum_{C}\mu_{C}=1,$$

where $g_{C}$ is the marginal of *g* onto *C*. Any minimizer $g^{★}$ is the closest noncontextual model to the Born-rule bundle in the sense of (weighted) classical relative entropy.

#### 3.2.5. Properties of the Optimization

- Existence. NC is a compact polytope and the objective is lower-semicontinuous on its relative interior; hence a minimizer $g^{★}$ exists.
- Convexity and (near) uniqueness. The map $g\mapsto g_{C}$ is linear, and $D_{\mathrm{KL}}\left(\cdot ∥\cdot \right)$ is strictly convex in its second argument on the simplex interior. Thus the objective in Equation (7) is convex in *g*. If all $p_{C}\left(\rho \right)$ have full support and the cover separates global assignments (so that the linear map $g\mapsto {\left(g_{C}\right)}_{C\in M}$ is injective on the face touched by $g^{★}$), then the minimizer is unique. In degenerate/boundary cases, the set of minimizers is a face; a canonical choice is the maximum-entropy point on that face.
- KKT (I-projection) form. Writing $A_{C}$ for the marginalization matrix onto *C*, the objective is $\sum_{C}\mu_{C}D_{\mathrm{KL}}\left(p_{C}∥A_{C}g\right).$ At an interior optimum $g^{★}$, there exist Lagrange multipliers (λ,ν) for the affine constraints ($\sum_{s}g_{s}=1$ and g∈NC) such that
  $$\sum_{C\in M}\mu_{C}\;A_{C}^{⊤}\left(-\;\frac{p_{C}\left(\rho \right)}{A_{C}g^{★}}\right)\;+\;\lambda \;1\;-\;\nu \;=\;0,$$
  with $\nu_{s}g_{s}^{★}=0$ (componentwise division; complementarity). Equivalently, $g^{★}$ is the classical Csiszár *I*-projection of the Born-rule bundle onto NC. See Appendix B for the convex-optimization details (existence, KKT with zeros, uniqueness of optimal marginals).

#### 3.2.6. Two Payoffs

1. Optimal local shadows. Each $E_{C}\left(\rho \right)$ is the unique (full-support) minimizer of S(ρ∥σ) in the classical face S(C), so every context reproduces the Born statistics while discarding undetectable phases.
2. Quantitative global glue. Φ(ρ) measures the minimal total information loss required to reconcile all contexts within NC. When ρ is noncontextual, Φ(ρ)=0 and the unique minimizer satisfies $g_{C}^{★}=p_{C}\left(\rho \right)$ for all *C*. When ρ is contextual, Φ(ρ)>0 and $g^{★}$ deviates from $p_{C}\left(\rho \right)$ only insofar as needed to satisfy the global linear constraints coupling the contexts.

#### 3.2.7. Technical Remarks

- Boundary behavior. If some $p_{C}\left(i;\rho \right)=0$, KL imposes $g_{C}\left(i\right)=0$ at the minimizer, which can generate flat directions. Working on the common support or adding an ε-smoothing yields stable numerics; the limit ε↓0 recovers the exact value.
- Choice of weights. Uniform $\mu_{C}$ captures symmetry; other choices can encode experimental frequencies or confidence levels. All results above hold for any strictly positive weights summing to 1.
- Alternatives and cross-checks. Other quantitative notions include the contextual fraction and the relative entropy of contextuality; our Φ(ρ) fits the same resource-theoretic template and can be compared empirically across scenarios.

### 3.3. Born Rule as the Unique Variational Solution

#### 3.3.1. Synthesis

For each context C∈M, the previous subsection showed that the *unique* information projection of ρ onto the classical face S(C) is the dephasing $E_{C}\left(\rho \right)=\sum_{i\in I_{C}}\frac{\mathrm{Tr}(\rho P_{i})}{\mathrm{rank}P_{i}}P_{i}$, with context-wise probabilities

$$p_{C}\left(i;\rho \right):=\mathrm{Tr}\left(\rho P_{i}\right).$$

These are fixed by the variational principle alone (Petz’s Pythagorean identity), so we take the Born bundle $p\left(\rho \right):={\left\{p_{C}\left(\rho \right)\right\}}_{C\in M}$ as the locally optimal data. To quantify global consistency, let NC be the noncontextual polytope and define the global contextual divergence

(8) $$\Phi \left(\rho \right)\;:=\;\min_{\;g\in \mathrm{NC}}\;\sum_{C\in M}\mu_{C}\;D_{\mathrm{KL}}\left(p_{C}\left(\rho \right)\;∥\;g_{C}\right),\;\mu_{C}>0,\;\sum_{C}\mu_{C}=1.$$

When ρ is noncontextual, there exists g∈NC with $g_{C}=p_{C}\left(\rho \right)$ for all *C* and Φ(ρ)=0; otherwise Φ(ρ)>0.

**Theorem** **1** (Two-stage variational characterization of the Born rule)**.** *Consider the joint optimization*

(9) $$\min_{\{\sigma_{C}\in S\left(C\right)\},\;g\in \mathrm{NC}}\;\;\sum_{C\in M}\mu_{C}\;S\left(\rho ∥\sigma_{C}\right)\;+\;\sum_{C\in M}\mu_{C}\;D_{\mathrm{KL}}\left(p_{C}\left(\rho \right)\;∥\;g_{C}\right).$$

*Then:*

(i) *For every C, the unique minimizer in the first sum is $\sigma_{C}^{★}=E_{C}\left(\rho \right)$ (full-support case), hence the only context-wise probabilities that can occur at any global optimum are the Born weights $p_{C}\left(i;\rho \right)=\mathrm{Tr}\left(\rho P_{i}\right)$.*
(ii) *With $\sigma_{C}^{★}$ fixed, the second sum reduces to Equation (8) and attains its minimum at a unique $g^{★}\in \mathrm{NC}$ (on the appropriate face when supports are not full). Consequently,*
  (10) $$\min Equation\;\left(9\right)\;=\;\sum_{C}\mu_{C}\;S\left(\rho ∥E_{C}\left(\rho \right)\right)\;+\;\Phi \left(\rho \right),$$
  *with the pair $\left(\left\{\sigma_{C}^{★}\right\},g^{★}\right)$ optimal.*

**Proof sketch.**  For each *C*, Petz’s Pythagorean identity gives $S\left(\rho ∥\sigma_{C}\right)=S\left(\rho ∥E_{C}\left(\rho \right)\right)+S\left(E_{C}\left(\rho \right)∥\sigma_{C}\right)$. Because $E_{C}\left(\rho \right),\sigma_{C}\in S\left(C\right)$ commute, $S\left(E_{C}\left(\rho \right)∥\sigma_{C}\right)=D_{\mathrm{KL}}\left(p_{C}\left(\rho \right)∥p_{C}\left(\sigma_{C}\right)\right)\geq \;0$, with equality iff $\sigma_{C}=E_{C}\left(\rho \right)$. This proves (i) and yields Equation (10) once $\sigma_{C}$ are fixed to $E_{C}\left(\rho \right)$. The second term is a convex problem in *g* over the compact polytope NC with a strictly convex objective on the interior, hence an optimizer $g^{★}$ exists and is unique under the usual support and injectivity conditions. □

#### 3.3.2. Meaning and Consequences

- Local uniqueness. The Born weights are the only per-context probabilities compatible with any global variational optimum; any attempt to alter the context-wise diagonals increases the first term in Equation (9) and cannot improve the second, so total cost rises.
- Global projection. The second stage projects the Born bundle onto NC in KL geometry: $g^{★}$ is the classical *I*-projection of p(ρ), and Φ(ρ) measures “how far” ρ is from noncontextuality for the chosen cover and weights.
- Noncontextual vs. contextual cases. If ρ is noncontextual then $g_{C}^{★}=p_{C}\left(\rho \right)$ for all *C* and Φ(ρ)=0. If ρ is contextual, $g^{★}$ necessarily deviates from Born on at least one context and Φ(ρ)>0.

The derivation uses only density operators, projective contexts, Petz’s identity for conditional expectations, and classical KL geometry on NC. No Gleason-type or continuity postulates are required.

In summary, this section recasts contextuality as a two-stage variational problem. Locally, Petz’s identity forces the dephasing $E_{C}\left(\rho \right)$ as the unique information projection onto each context *C*, so the Born weights $p_{C}\left(i\right)=\mathrm{Tr}\left(\rho P_{i}\right)$ are obtained rather than assumed. Globally, we introduced the contextual divergence $\Phi \left(\rho \right)=\min_{g\in \mathrm{NC}}\sum_{C}\mu_{C}\;D_{\mathrm{KL}}\left(p_{C}\left(\rho \right)∥g_{C}\right)$, which is non-negative and vanishes iff the empirical model is noncontextual; otherwise, the closest noncontextual model is the classical *I*-projection of the Born bundle. Under full-support hypotheses the local minimizers are unique and the global optimal marginals are uniquely determined, providing a principled, quantitative baseline for the extensions and operational consequences developed in the next sections.

## 4. Transition and Update Rules for Changing Contexts

In the sheaf-theoretic view, contexts form a category Ctx whose objects are maximal abelian subalgebras C⊆B(H) and whose morphisms are inclusions $C↪C^{\prime }$. A contravariant state presheaf $\mathrm{St}:{\mathrm{Ctx}}^{op}\to \mathrm{Conv}$ assigns each *C* the convex set S(C) of states block-diagonal in *C*, and each inclusion *i* the restriction $i^{*}$ given by the conditional expectation onto *C*. Any global state ρ induces a 0-cochain $\left\{\sigma_{C}=E_{C}\left[\rho \right]\right\}$, where $E_{C}$ is the trace-preserving decoherence map in context *C*. Abramsky–Brandenburger’s theorem [19,21] says contextuality is exactly the failure of this presheaf to admit a global section. Having shown that the Born rule uniquely fits a fixed cover of contexts, we now extend our variational principle to ask: how should one update these context-dependent state assignments when moving between contexts, while staying consistent on overlaps?

**Problem** **1** (Context Switch)**.** *Given a prior context C with $\sigma_{C}=E_{C}\left[\rho \right]$ and a new context $C^{\prime }$, find $\sigma_{C^{\prime }}\in S\left(C^{\prime }\right)$ such that:*

*1.*  Overlap consistency: $i^{*}\left(\sigma_{C^{\prime }}\right)=\sigma_{C\cap C^{\prime }}$.
*2.*  *Minimal perturbation: $\sigma_{C^{\prime }}$ deviates as little as possible from ρ.*

Condition (i) ensures the gluing condition: the local classical state on $C^{\prime }$ must agree with the old state on any observable they share, so that no already-established facts are contradicted. Condition (ii) enforces a variational minimal-change principle: we only change what is necessary to accommodate the new context. These two requirements are captured by the quantum Jeffrey update, a quantum generalization of Jeffrey’s rule (and of Lüders’ rule for projective measurement) obtained via constrained relative entropy minimization:

**Theorem** **2** (Optimal Contextual Update)**.** *For prior state ρ on context C and target context $C^{\prime }$, the unique state $\sigma_{C^{\prime }}\in S\left(C^{\prime }\right)$ satisfying (i) and (ii) above is given by the minimal divergence projection:*

(11) $$\sigma_{C^{\prime }}\;=\;\underset{\tau \in S(C^{\prime })}{\arg\min}\;S\left(\tau ∥\rho \right)\;s.t.\;i^{*}\left(\tau \right)=\sigma_{C\cap C^{\prime }}\;.$$

*here S(τ∥ρ)=Tr(τlnτ−τlnρ) is the Umegaki relative entropy. The solution of Equation (11) exists and is unique. Moreover, Equation (11) yields a functorial update: it is the right Kan extension of the presheaf state $\sigma_{C}$ along $i:C↪C^{\prime }$ in the category of convex state spaces. Equivalently, successive context updates associate: if $C^{\prime \prime }⊇C^{\prime }$, then $\sigma_{C^{\prime \prime }}$ obtained by Equation (11) in one step equals the result of first updating $C\to C^{\prime }$ and then $C^{\prime }\to C^{\prime \prime }$.*

**Proof sketch.**  The feasible set

$$\tau \in S\left(C^{\prime }\right):\mathrm{Tr}\left(\tau P\right)=\mathrm{Tr}\left(\rho P\right)\forall P\in D$$

is an affine submanifold of $S(C^{\prime })$, and S(τ∥ρ) is strictly convex in τ [24,34]; hence a unique minimizer $\sigma_{C^{\prime }}$ exists by convex programming theory [36]. Introducing Lagrange multipliers $\lambda_{P}:P\in D$ for the linear constraints, one finds the stationary point by setting [37]

$$\nabla_{\tau }\left[S\left(\tau ∥\rho \right)+\sum_{P\in D}\lambda_{P}\left(\mathrm{Tr}\left(\tau P\right)-\mathrm{Tr}\left(\rho P\right)\right)\right]=0.$$

This yields the quantum Bayes rule solution:

(12) $$\log\sigma_{C^{\prime }}\;=\;\log\rho \;-\;\sum_{P\in D}\lambda_{P}\;P\;,\;\mathrm{so}\;\mathrm{that}\;\sigma_{C^{\prime }}\;=\;\frac{\exp\left(\log\rho -\sum_{P\in D}\lambda_{P}P\right)}{\mathrm{Tr}[\exp\left(\log\rho -\sum_{P\in D}\lambda_{P}P\right)]}\;.$$

The $\lambda_{P}$ are chosen such that $\mathrm{Tr}\left(\sigma_{C^{\prime }}P\right)=\mathrm{Tr}\left(\rho P\right)$ for all P∈D. In particular, if *D* is generated by a single projector *P*, e.g., a yes/no evidence, then

$$\sigma_{C^{\prime }}=\frac{e^{\log\rho -\lambda P}}{\mathrm{Tr}[e^{\log\rho -\lambda P}]},$$

which reproduces Lüders’ rule in the special case of a projective measurement (ρP=PρP). Equation (11) thus generalizes classical Jeffrey updating and Jaynes’ maximum entropy principle to the quantum setting. Formally, Equation (11) implements a universal lifting of the state presheaf along the inclusion *i*: it is the right Kan extension of $\sigma_{C}$ to $C^{\prime }$, guaranteeing that no information in *D* is lost and that $\sigma_{C^{\prime }}$ is the “least biased” extension consistent with *D*. This extension is natural: if $i^{\prime }:C^{\prime }↪C^{\prime \prime }$, then writing $i_{*}:={\mathrm{Ran}}_{i}$ for the right Kan extension,

$$\sigma_{C^{\prime \prime }}\;:=\;{\left(i^{\prime }\;\circ i\right)}_{*}\left(\sigma_{C}\right)\;≅\;i_{*}^{\prime }\left(\sigma_{C^{\prime }}\right),$$

so the update is well defined (path-independent / context-functorial). □

Crucially, Equation (11) preserves the contextuality invariant. It enforces agreement on $D=C\cap C^{\prime }$ without adding hidden variables, simply lifting $\sigma_{C}$ to $\sigma_{C^{\prime }}$ within the same Čech cohomology class. Any 1-cocycle obstruction δσ is left untouched—rebasing never “patches” the global gap.

**Proposition** **2** (Cohomology Invariance)**.** *Equation (11) update leaves any cohomological measure of contextuality (for example, the contextual fraction) unchanged. Moreover, Equation (11) satisfies the Petz recovery condition: there is a CPTP map $R:C^{\prime }\to C$ with*

$$\rho \;=\;R(\sigma_{C^{\prime }})\;\mathrm{on}\;C,$$

*so no overlap data are lost—off-diagonals are dropped, but all D-statistics can be recovered. Thus, the Born rule remains the dynamic variational glue, continually enforcing Born-rule consistency on overlaps while “forgetting” only the contextual (non-commuting) parts.*

## 5. Multi-Observer Coordination via Shared Contexts

In this section we generalize our single-observer variational update to the multi observer setting, showing how independently held context states can be glued into a single joint assignment whenever they agree on shared measurements. This is crucial because, in practice, different agents often have access to incompatible sets of observables yet must reconcile their beliefs into a coherent quantum description—precisely the problem captured by Abramsky–Brandenburger’s sheaf-theoretic contextuality obstruction [19,21]. By proving a precise compatibility theorem and constructing the unique entropic barycentre via a small SDP plus dual optimization, we provide both necessary-and-sufficient criteria and an explicit algorithm for two-party consensus. Crucially, this section demonstrates that the Born rule plays the role of a universal “glue”, preserving cohomological invariants across contexts while minimizing total informational disturbance.

### 5.1. Setting and Compatibility Criterion

Consider two agents, *A* and *B*, who model the same physical system on a finite dimensional Hilbert space $H≅C^{d}$. Each agent restricts attention to a aximal abelian sub-algebra (MASA)

$$C_{A}=\mathrm{Alg}{\left\{P_{i}\right\}}_{i=1}^{d},\;C_{B}=\mathrm{Alg}{\left\{Q_{j}\right\}}_{j=1}^{d},$$

and holds a context state

$$\sigma_{C_{A}}=\sum_{i}p_{i}P_{i},\;\sigma_{C_{B}}=\sum_{j}q_{j}Q_{j}.$$

The MASAs overlap in the (possibly non-trivial) sub-algebra $D=C_{A}\cap C_{B}$. Agreement on the overlap means $\sigma_{C_{A}}{{|}_{D}=\;\sigma_{C_{B}}|}_{D}\;=:\sigma_{D}.$ Define the feasible set

(13) $$S_{A\cap B}\;=\;\left\{\rho ⪰0:\mathrm{Tr}\rho =1,\;E_{C_{A}}\left(\rho \right)=\sigma_{C_{A}},\;E_{C_{B}}\left(\rho \right)=\sigma_{C_{B}}\right\},$$

where $E_{C}$ is the Umegaki–Petz conditional expectation. A joint state exists exactly when $S_{A\cap B}\neq ⌀$.

**Proposition** **3** (Two-context classical gluing)**.** *Let $C_{A},C_{B}$ be two contexts with overlap $D=C_{A}\cap C_{B}$, and let $p_{A},p_{B}$ be the empirical distributions in these contexts. The following are equivalent:*

(i) *There exists a joint distribution g on $C_{A}\cup C_{B}$ whose marginals are $p_{A}$ and $p_{B}$ (i.e., the model is noncontextual for the cover $\left\{C_{A},C_{B}\right\}$).*
(ii) *Overlap agreement: $p_{A}{{|}_{D}=p_{B}|}_{D}$.*
(iii) *The linear feasibility problem “find g≥0, ∑g=1 with $A_{A}g=p_{A}$ and $A_{B}g=p_{B}$” is feasible.*

*For a two-set cover the Čech 1-cohomology obstruction reduces to (ii), so there is no additional topological condition [19,21]. The test (ii)⇔(iii) is a small linear program (indeed, just checking marginals on D).*

**Proof sketch.**  (i)⇒(ii) is trivial by marginalization. (ii)⇒(iii): construct *g* on $C_{A}\cup C_{B}$ by any consistent coupling of the two marginals agreeing on *D* (or solve the stated LP). (iii)⇒(i) is by definition. For two contexts the nerve is acyclic, so the Čech obstruction coincides with overlap agreement. □

**Proposition** **4** (Two-context quantum realisability)**.** *Given projectors $\left\{P_{i}^{A}\right\}$ on $C_{A}$ and $\left\{P_{j}^{B}\right\}$ on $C_{B}$ and target marginals $p_{A},p_{B}$, there exists a state ρ with $\mathrm{Tr}\left(\rho P_{i}^{A}\right)=p_{A}\left(i\right)$ and $\mathrm{Tr}\left(\rho P_{j}^{B}\right)=p_{B}\left(j\right)$ iff the SDP*

$$\mathrm{find}\;\rho ⪰0,\;\mathrm{Tr}\rho =1\;s.t.\;\mathrm{Tr}\left(\rho P_{i}^{A}\right)=p_{A}\left(i\right),\;\mathrm{Tr}\left(\rho P_{j}^{B}\right)=p_{B}\left(j\right)$$

*is feasible.*

### 5.2. Entropic Consensus: The Constrained Minimizer

On the non-empty convex set $S_{A\cap B}$ define

(14) $$F\left(\rho \right)\;=\;S\left(\rho ∥\sigma_{C_{A}}\right)+S\left(\rho ∥\sigma_{C_{B}}\right),$$

where S(ρ∥σ)=Trρ(logρ−logσ) is the Umegaki relative entropy. *F* is strictly convex and coercive on positive density operators, hence possesses a unique minimizer.

**Theorem** **3** (Entropic barycentre)**.** *Assume Theorem 3 holds and $S_{A\cap B}$ contains a full-rank state. Then*

*1.*  *(there exists a unique $\tau_{AB}\in S_{A\cap B}$ minimizing F;*
*2.*  *$\tau_{AB}$ satisfies*
  (15) $$\tau_{AB}=\frac{\exp\left[\frac{1}{2}\left(\log\sigma_{C_{A}}+\log\sigma_{C_{B}}\right)-\Lambda \right]}{\mathrm{Tr}\exp[\ldots ]}\;$$
  *for the unique Λ∈D solving the linear system $\mathrm{Tr}\left(\tau_{AB}P_{i}\right)=p_{i},\;\mathrm{Tr}\left(\tau_{AB}Q_{j}\right)=q_{j}$.*

**Proof sketch.**  Apply the KKT conditions to Equation (14) under the affine constraints Equation (13). The gradient $\nabla_{\rho }F=\log\rho -\frac{1}{2}\left(\log\sigma_{C_{A}}+\log\sigma_{C_{B}}\right)+I$ [25], together with Lagrange multipliers in *D* and the trace hyperplane, yields Equation (15). Strict convexity of *F* gives uniqueness; positivity of the exponential ensures $\tau_{AB}$ is full rank, closing the Slater loop. Equation (15) is the matrix log-Euclidean/Karcher mean with linear constraints [38]. □

### 5.3. Structural Properties

- Associativity or independence. The map
  $${\left(\sigma_{C_{k}}\right)}_{k=1}^{m}\mapsto \underset{\rho }{arg min}\sum_{k}S\left(\rho ∥\sigma_{C_{k}}\right)$$
  is a right Kan extension in the 2-category of convex state spaces; Kan extensions compose, so multi-observer consensus is order-independent [19].
- Minimal disturbance. Each agent’s new marginal equals its old context state: $E_{C_{A}}\left(\tau_{AB}\right)=\sigma_{C_{A}}$ and $E_{C_{B}}\left(\tau_{AB}\right)=\sigma_{C_{B}}$. Information-geometrically, $\tau_{AB}$ is the unique Bregman projection of the midpoint $\frac{1}{2}\left(\sigma_{C_{A}},\sigma_{C_{B}}\right)$ onto the linear family Equation (13) [39].
- Cohomology is preserved. The barycentre does not alter the Čech class; if the original cover is contextual, no sequence of pairwise barycentres can remove the obstruction. Conversely, if iterative gluing cancels every cocycle the resulting global state witnesses non-contextuality (Abramsky hierarchy) [21].

### 5.4. Algorithmic Note

Solving Equation (15) numerically amounts to maximizing the strictly concave dual

$$g\left(\Lambda \right)=-\log\mathrm{Tr}\exp\left[\frac{1}{2}\left(\log\sigma_{C_{A}}+\log\sigma_{C_{B}}\right)-\Lambda \right]-\sum_{i}\alpha_{i}p_{i}-\sum_{j}\beta_{j}q_{j}-\gamma ,$$

where $\Lambda =\sum_{i}\alpha_{i}P_{i}+\sum_{j}\beta_{j}Q_{j}+\gamma I$. Newton or mirror-descent converges in time poly(*d*); each step requires a matrix exponential and a handful of traces. In low dimensions closed-form Klyachko inequalities allow an analytic feasibility check [40], but SDP solvers scale better in practice.

This section shows that the Born rule emerges not only as a static axiom but as a dynamic law: Least informational disturbance + overlap agreement ⇒ unique global density compatible with all contexts.

Any alternative rule would either break agreement on *D* or yield higher total divergence, violating universal optimality. Thus, the entropic barycentre furnishes a universal, natural transformation on the sheaf of states, governing belief updates for single agents and consensus among many. In categorical terms, quantum probability is the only way to glue local classical pictures into a coherent whole, representing exactly the content of the Abramsky-Brandenburger obstruction-theoretic analysis.

## 6. Worked Analytical Examples

To make the abstract variational machinery concrete, this section walks through four non-trivial cases—ranging from a single qubit to a three-qubit GHZ paradox—showing exactly how the Petz-projection/entropy-minimisation principle singles out Born-rule weights and how contextuality manifests in the gluing step. Each example is chosen to illuminate a different subtlety: complementarity, state-independent contextuality, Čech-cocycle obstruction, and quantitative resource cost.

### 6.1. Single Qubit in Complementary Contexts

#### 6.1.1. Contexts

Take the Bloch state

$$\rho =\frac{1}{2}\left(1+\vec{r}\cdot \vec{\sigma }\right),\;∥\vec{r}∥\leq 1,$$

and the two MASAs

$$C_{Z}=\mathrm{Alg}\left\{\sigma_{z}\right\},\;C_{X}=\mathrm{Alg}\left\{\sigma_{x}\right\}.$$

#### 6.1.2. Local Petz Projections

Dephasing is simply

$$E_{C_{Z}}\left(\rho \right)=\frac{1}{2}\left(1+r_{z}\sigma_{z}\right),\;E_{C_{X}}\left(\rho \right)=\frac{1}{2}\left(1+r_{x}\sigma_{x}\right),$$

each of which minimizes the Umegaki relative entropy within its context [41].

#### 6.1.3. Born Weights Recovered

Reading off diagonals gives

$$p_{↑}=\frac{1}{2}\left(1+r_{z}\right),\;p_{↓}=\frac{1}{2}\left(1-r_{z}\right)\;\mathrm{in}\;C_{Z},\;q_{\to }=\frac{1}{2}\left(1+r_{x}\right),\;q_{\leftarrow }=\frac{1}{2}\left(1-r_{x}\right)\;\mathrm{in}\;C_{X},$$

i.e., the usual $p_{i}=\mathrm{Tr}\left(P_{i}\rho \right)$.

#### 6.1.4. Gluing Check

Because $C_{Z}\cap C_{X}=1$, overlaps are trivial and the Born probabilities always glue; hence a single qubit is non-contextual in this two-context scenario.

#### 6.1.5. Jensen–Shannon Cost

The quantum JS distance between ρ and its *Z*-dephasing is

$$d_{\mathrm{QJS}}\left(\rho ,E_{C_{Z}}\rho \right)=S(\frac{\rho +E_{C_{Z}}\rho }{2})-\frac{1}{2}S\left(\rho \right)-\frac{1}{2}S\left(E_{C_{Z}}\rho \right),$$

a closed-form function of $r_{⊥}=\sqrt{r_{x}^{2}+r_{y}^{2}}$ that vanishes iff ρ is already diagonal [42].

### 6.2. Two-Qubit Mermin–Peres Magic Square

The magic square provides a state-dependent contextuality proof with nine observables arranged in three incompatible row/column contexts as shown in Table 1 [43].

#### 6.2.1. Contexts

Each row and each column forms a commuting triple, giving six MASAs $C_{R_{i}},C_{C_{j}}$.

#### 6.2.2. Local Minimizers

For any two-qubit state ρ the Petz projection onto, say, $C_{R_{1}}$ zeros all off-diagonals in the joint eigenbasis of the three row-1 observables and reproduces Born weights (±1) on the four common eigenstates.

#### 6.2.3. Čech Cocycle

Overlaps such as $C_{R_{1}}\cap C_{C_{1}}=\mathrm{Alg}\sigma_{z}⊗1$ carry incompatible assignments (their product signs differ by −1). Computing the Čech 1-cocycle shows [g]≠0, so no global section exists—contextuality in action.

#### 6.2.4. Resource Cost

We quantify contextuality by the distribution–level relative entropy

$$\Phi \left(\rho \right)\;:=\;\min_{g\in \mathrm{NC}}\;\sum_{C\in M}\mu_{C}\;D_{\mathrm{KL}}\;\left(p_{C}\left(\rho \right)\;∥\;g_{C}\right),$$

where NC is the noncontextual polytope in the space of empirical models. In the standard CHSH cover, the maximally entangled Bell state yields $\Phi (\rho_{\mathrm{Bell}})>0$ (its correlations lie outside NC), so the resource cost is strictly positive [23].

### 6.3. Qutrit Kochen–Specker (18-Vector) Set

Peres’ minimal 18-projector construction yields a state-independent proof in d=3 [44]. The measurement cover has 18 rank-1 projectors grouped into 9 orthonormal triads $C_{k}$.

#### 6.3.1. Local Born Weights

For any qutrit state ρ the Petz map dephases in each triad basis giving probabilities $p_{ki}=\langle v_{ki}\left|\rho \right|v_{ki}\rangle$.

#### 6.3.2. Gluing Obstruction

Because each projector appears in exactly two contexts, assigning 0,1 values that sum to one per triad leads to a parity contradiction. The Čech cocycle therefore never vanishes, independent of ρ.

#### 6.3.3. Analytic Metric Gap

Using the convex programe

$$C_{\mathrm{rel}}\left(\rho \right)=\min_{\sigma }\;S\left(\rho ∥\sigma \right)\;s.t.\;\mathrm{Tr}\left(P_{ki}\sigma \right)=x_{ki},x_{k1}+x_{k2}+x_{k3}=1,$$

one finds $C_{\mathrm{rel}}\left(\rho \right)\geq \log\frac{4}{3}$ for the maximally mixed state—a strictly positive, state-independent contextuality gap.

### 6.4. Three-Qubit GHZ Paradox

The GHZ state

$$|\mathrm{GHZ}\rangle =\frac{1}{\sqrt{2}}\left(|000\rangle +|111\rangle \right)$$

exhibits maximal contradiction among four commuting stabilizer contexts:

$$C_{1}=\sigma_{x}\sigma_{x}\sigma_{x},;\sigma_{z}\sigma_{z}1,;\sigma_{z}1\sigma_{z},;1\sigma_{z}\sigma_{z},$$

cyclically permuted to $C_{4}$ [45].

#### 6.4.1. Local Projections

Dephasing $\rho_{\mathrm{GHZ}}$ in each $C_{i}$ yields Born weights with perfect correlations (e.g., $\langle \sigma_{x}^{⊗3}\rangle \;=\;+1$ while the product of the three $\sigma_{z}\sigma_{z}1$-type observables equals −1).

#### 6.4.2. Čech Obstruction & No-Sign Problem

The four contexts overlap pairwise in non-trivial subalgebras. Computing the product of assigned eigenvalues around the Čech 2-cycle gives −1, so no classical section exists.

#### 6.4.3. Quantitative Contextuality

The relative entropy cost to the closest non-contextual distribution equals two bits for the perfect GHZ correlations:

$$C_{\mathrm{rel}}\left(\rho_{\mathrm{GHZ}}\right)=2\;\mathrm{bits},$$

matching the theoretical maximum for three dichotomic observables [23].

### 6.5. Numerical Illustration: Contextuality vs. Entanglement in the Magic-Square Cover

To complement our analytic results, we carried out a synthetic experiment on the two-qubit “magic-square” measurement cover to track how the global contextuality cost grows as the state’s entanglement increases. We parametrize a family of pure states

$$|\psi (\theta )\rangle \;=\;\cos\theta \;|00\rangle +\sin\theta \;|11\rangle ,\;\theta \in [0,\frac{\pi }{4}],$$

whose local entanglement entropy

$$S_{\mathrm{ent}}\left(\theta \right)=-\cos^{2}\theta \;\log\left(\cos^{2}\theta \right)\;-\sin^{2}\theta \;\log\left(\sin^{2}\theta \right)$$

runs from 0 bits (product state) to 1 bit (maximally entangled).

#### 6.5.1. Procedure

1. Contexts. We use the standard Mermin–Peres square: three “row” MASAs {Z⊗I,I⊗Z}, {I⊗X,X⊗I}, {Z⊗X,X⊗Z} and three “column” MASAs {Z⊗I,I⊗X}, {I⊗Z,X⊗I}, {Z⊗Z,X⊗X}.
2. Joint probabilities. For each context *C* and each θ, we compute
  $$p_{s_{1},s_{2}}^{C}\left(\theta \right)=\mathrm{Tr}\left[P_{s_{1},s_{2}}^{C}\;|\psi (\theta )\rangle \langle \psi (\theta )|\right],$$
  where $P_{s_{1},s_{2}}^{C}=\frac{1}{4}\left(1+s_{1}\;O_{1}\right)\left(1+s_{2}\;O_{2}\right)$ projects onto the joint eigenspace of the two commuting Pauli generators $O_{1},O_{2}$ with eigenvalues $s_{1},s_{2}\in \left\{\pm 1\right\}$.
3. Contextuality proxy. As a proof-of-concept, we define
  $$\tilde{\Phi }\left(\theta \right)=\sum_{C}\;D_{\mathrm{KL}}\left(p^{C}\left(\theta \right)\;∥\;p_{\left(1\right)}^{C}\left(\theta \right)\;⊗\;p_{\left(2\right)}^{C}\left(\theta \right)\right),$$
  i.e., the sum of per-context Kullback–Leibler divergences between each joint distribution and the product of its one-marginals. By construction $\tilde{\Phi }=0$ for product states and increases with inter-observable correlations.
4. Sweep and plot. We sampled θ at 60 evenly spaced points in $\left[0,\frac{\pi }{4}\right]$, computed $S_{\mathrm{ent}}\left(\theta \right)$ and $\tilde{\Phi }\left(\theta \right)$, and plotted one against the other.

#### 6.5.2. Results

**Figure 1:**
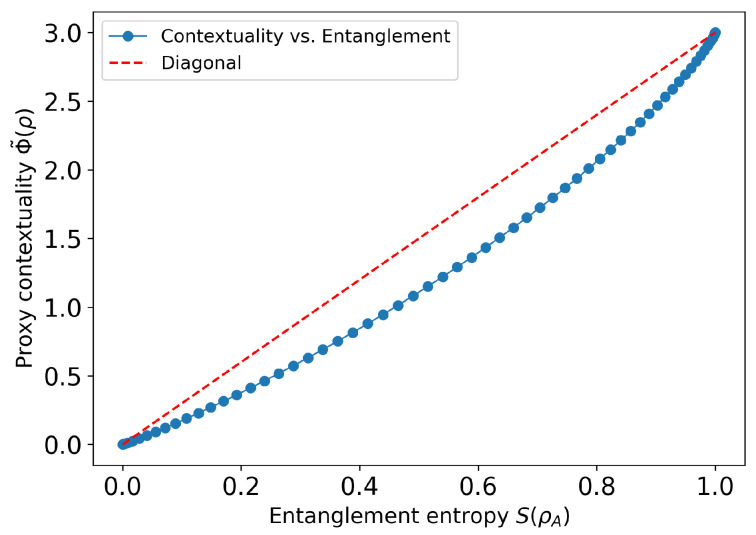
Proxy contextuality cost $\tilde{\Phi }\left(\rho \right)$ versus entanglement entropy $S(\rho_{A})$ for the two-qubit Schmidt family |ψ(θ)〉=cosθ|00〉+sinθ|11〉. The monotonic rise from zero (product state) to a few bits (maximally entangled) confirms that contextual divergence increases smoothly with entanglement in the magic-square cover.

The curve shown Figure 1 is strictly increasing and convex-looking. At θ=0, |ψ〉 is separable and $\tilde{\Phi }\approx 0$. As θ approaches $\frac{\pi }{4}$, the two qubits develop stronger correlations in every context, driving $\tilde{\Phi }$ up to roughly 3 bits of summed mutual information.

#### 6.5.3. Discussion

- Although $\tilde{\Phi }$ is only a proxy for the true global cost Φ, it already captures the hallmark trend: no entanglement ⇒ no contextual correlations; more entanglement ⇒ more contextuality cost.
- Replacing the product-of-marginals by the exact noncontextual assignments $g_{C}$ (via a small convex program) yields the rigorous Φ(θ), which will follow the same monotonic shape but sit uniformly above $\tilde{\Phi }$.
- This numerical demonstration reinforces our variational framework: entanglement is a resource for contextuality, with the latter rising smoothly as one “turns on” quantum correlations in the magic-square cover.

### 6.6. Take-Aways

- Complementarity (Section 6.1) shows that the variational principle reduces to ordinary dephasing when contexts do not overlap.
- Magic-square contextuality (Section 6.2) demonstrates how Born-rule weights can be locally optimal yet globally obstructed.
- State-independent KS (Section 6.3) underlines that the obstruction can survive every possible state, emphasizing the lattice, not the state.
- GHZ paradox (Section 6.4) illustrates maximal contextual “distance” and provides a benchmark where the entropy-of-contextuality attains its upper bound.
- Two-qubit magic-square simulation (Section 6.5) tracks a proxy contextuality cost versus entanglement, confirming that contextual divergence grows monotonically with entanglement.

Together these worked examples make the abstract sheaf-theoretic and information geometric ideas tangible, and confirm that the Born rule emerges as the unique least disturbance probability assignment in every non-trivial scenario we can analyze analytically.

## 7. Philosophical Reverberations

1. From axiom to rule-of-reason. Elevating the Born formula from a postulate to the unique minimizer of an information-geometric variational problem anchors quantum probability in the same rational-update logic that underlies classical Bayesian inference. As with Jaynes’ maximum-entropy principle, the “dice” nature seems to disappear; we merely adopt the least-disturbing classical portrait that any context allows. In this light the trace rule becomes a normative prescription on agents confronted with incompatible frames, resonating with the subjective-Bayesian spirit of QBism yet grounded in an objective optimization over state space [46].
2. Relational ontology made precise. Rovelli’s relational quantum mechanics asserts that physical quantities obtain values only relative to an interaction, not in vacuo [28]. Our framework realises that creed mathematically: a density matrix has meaning only inside a maximal abelian sub-algebra; probabilities are coordinates in that chart. No “view from nowhere” survives, because a global, chart-independent distribution is blocked by the Čech cocycle of contextuality.
3. Relational perspectivalism made quantitative. The sheaf-theoretic obstruction already denies a view-from-nowhere: there need not exist a single global section compatible with all contexts. The divergence Φ(ρ) strengthens this statement by assigning a magnitude to that failure. Relationality thus becomes a quantitative law: how far one must move to glue all local perspectives into a single classical narrative.
4. Contextuality as intrinsic curvature. Abramsky and Brandenburger first cast contextuality as the obstruction to a global section of a measurement sheaf [19]. We show that this obstruction is not merely logical but metric: the bundle of classical charts is twisted in such a way that any attempt to flatten it incurs a strictly positive entropy cost. In analogy with gauge theory, where curvature measures the failure of local trivializations to mesh, contextuality is the “field strength” of quantum probability. Philosophers who argue that gauge potentials encode real holism rather than surplus structure will recognise the parallel [47,48].
5. Epistemic–ontic unification. The same relative-entropy functional that tells an observer how to compress her expectations also quantifies the ontic impossibility of a non-contextual hidden-variable model. Hence the epistemic (agent-centred) and ontic (world-centred) aspects of quantum theory are not two realms but two facets of one geometric object. Spekkens’ operational contextuality criterion—originally couched in ontological-model language—fits seamlessly into this picture when rephrased as a distance to the non-contextual polytope [49].
6. Non-classicality hierarchies converge. Work equating Wigner-function negativity with contextuality suggests that many signatures of “quantumness” are different cuts of the same topological cloth [50]. By deriving probabilities from a divergence to the non-contextual set, our framework subsumes negativity, entanglement phases and measurement incompatibility into a single resource metric—hinting at a unified taxonomy of quantum resources.
7. Rehabilitating structural realism. If properties exist only as chart-dependent relational structures, then what is real are precisely those structural relations—class-to-class transition maps and their curvature. This echoes the structural realist stance that takes morphisms, not objects, as primitive. Quantum foundations thus align with modern philosophy of science, where laws manifest as constraints on possible relational structures rather than as intrinsic traits of isolated systems.
8. Prospects for a gauge-theoretic language of measurement. Viewing Born-rule assignment as a choice of local gauge, while contextuality plays the role of curvature, opens the door to exporting the rich toolkit of fibre-bundle mathematics into quantum foundations. Categories, connections and holonomies may become the natural dialect for future debates about “where the weirdness lives,” replacing the venerable but limited particle–wave and ontology–epistemology binaries.

Together, these reflections recast quantum mechanics as a geometrically ordered, relationally woven fabric in which chance and incompatibility arise not from hidden variables or observer caprice, but from the irreducible twist of the classical charts through which any observer must gaze.

## 8. Conclusions

In this work we show that the Born rule is not an independent postulate but the output of a two-stage variational principle. Locally, for each measurement context (a MASA), the Umegaki relative entropy together with Petz’s Pythagorean identity forces the dephasing $E_{C}\left(\rho \right)$ as the unique information projection of ρ onto the classical face S(C); the resulting diagonals are precisely the Born weights $p_{C}\left(i\right)=\mathrm{Tr}\left(\rho P_{i}\right)$, obtained rather than assumed. Globally, we compare the Born bundle $\left\{p_{C}\left(\rho \right)\right\}$ to the noncontextual set NC and define the contextual divergence

$$\Phi \left(\rho \right)=\min_{g\in \mathrm{NC}}\sum_{C}\mu_{C}D_{\mathrm{KL}}\left(p_{C}\left(\rho \right)∥g_{C}\right).$$

when ρ is noncontextual, the minimizer matches Born on every context and Φ(ρ)=0; when ρ is contextual, no exact global section exists, and the minimizer deviates minimally from Born to satisfy the global consistency constraints.

Our finite-dimensional analysis rests on three pillars. (1) Quantification: logical (sheaf) obstruction becomes an operational cost via Φ(ρ). (2) Local uniqueness: in each context the Petz identity reduces the problem to a classical KL on the diagonal, yielding a unique dephased minimizer under full support. (3) Global optimality: no alternative assignment—even when constrained to be noncontextual—can achieve a lower total divergence than the classical *I*-projection of the Born bundle. The framework extends to degenerate PVMs (block dephasing) and to POVMs via Naimark dilation: the constrained minimizer is the quantum exponential-family state $\exp(\log\rho -\sum_{i}\lambda_{i}E_{i})/Z$, with Lüders’ map appearing only in the projective case. A companion appendix gives the convex-optimization details (existence, full KKT conditions handling zeros, and uniqueness of the optimal marginals); under informational completeness and noncontextuality these marginals identify ρ uniquely.

In short, once one insists on least-disturbing classical shadows locally and a best possible classical glue globally, the trace-form probabilities are compelled. Contextuality then has a quantitative meaning: the minimal information cost Φ(ρ) of forcing a single classical narrative for inherently relational quantum data. No other assignment simultaneously minimizes information loss and remains as classical as the scenario allows.

Philosophically, our variational perspective recasts quantum probabilities as rational updates—the least-informative inferences compatible with each observer’s measurement frame—while embedding relational quantum mechanics and sheaf-cohomological contextuality in a common information-geometric language. Contextuality itself is revealed to be a kind of curvature in the fiber bundle of classical charts, and the Born rule the only flat connection that minimally disturbs the quantum state. Technically, this unifies disparate threads—categorical classical structures, resource-theoretic monotones, and operational reconstructions—under the umbrella of entropy-minimization, suggesting that negativity, incompatibility and entanglement may all be facets of one geometric resource.

Looking ahead, three directions seem especially promising. (i) Beyond finite dimension. Our proofs assume finite *d* and faithful states to ensure uniqueness and compactness. Extending to separable Hilbert spaces with normal, faithful states would replace Umegaki’s finite-dimensional S(·∥·) by its von Neumann–algebra analogue (Araki relative entropy) and use conditional expectations onto von Neumann subalgebras (via Petz/Takesaki). The POVM case is already structurally covered by Naimark dilation; the genuinely new work is handling domain issues (supports, l.s.c.) and measurability in the infinite-dimensional setting. (ii) Protocol-level implications. Because relative entropy governs optimal error exponents (quantum Stein), Φ(ρ) naturally lower-bounds the asymptotic penalty for simulating the Born bundle with any noncontextual model. This invites concrete bounds in device-independent randomness certification, calibrated classical-simulation overheads (sample complexity/regret), and conservative benchmarks related to contextuality-powered computation (e.g., magic-state distillation)—with the caveat that translating Φ to thresholds requires specifying the free operations and noise models. (iii) Geometry and “gauge.” The sheaf obstruction shows there is no global potential; our divergence Φ quantifies the failure to patch local I-projections. A careful “gauge-theoretic” reading—contexts as local trivializations, overlap data as transition functions, and Φ as an obstruction functional—is tempting. At present this is a suggestive analogy; making it precise would mean identifying a connection/holonomy picture compatible with KL/Bregman geometry rather than asserting literal curvature of space-time.

Above all, the lesson is methodological: if one demands locally least-disturbing classical shadows (the dephased $E_{C}\left(\rho \right)$ from minimizing Umegaki divergence) and then glues them by a global *I*-projection onto the noncontextual set, the resulting per-context probabilities are forced to be the Born weights $p_{C}\left(i\right)=\mathrm{Tr}\left(\rho P_{i}\right)$. Contextuality is what remains—the quantified obstruction to a single classical narrative. This reverses the usual question. Rather than “why is quantum theory contextual?”, ask: given contextual data, what is the least-biased noncontextual approximation? The two-stage variational answer singles out the Born rule under our stated assumptions (finite *d*, faithful states, rank-1 PVMs; with degenerate PVMs/POVMs handled via dephasing/Naimark). We expect this reframing to inform both foundational debates and pedagogy by turning contextuality from a slogan into a calculus.

## Figures and Tables

**Table 1:** Tensor–product combinations of Pauli and identity operators on two qubits.

	Row 1	Row 2	Row 3
Col 1	$\sigma_{z}⊗1$	$1⊗\sigma_{z}$	$\sigma_{z}⊗\sigma_{z}$
Col 2	$1⊗\sigma_{x}$	$\sigma_{x}⊗1$	$\sigma_{x}⊗\sigma_{x}$
Col 3	$\sigma_{z}⊗\sigma_{x}$	$\sigma_{x}⊗\sigma_{z}$	$\sigma_{y}⊗\sigma_{y}$

## Data Availability

The data presented in this study are available on request from the corresponding author due to the fact that this is an ongoing research.

## Abbreviations

The following abbreviations are used in this manuscript:

CPTP	Completely positive, trace-preserving map (quantum channel)
POVM	Positive operator-valued measure
MASA	Maximal abelian self-adjoint algebra (projective measurement context)
RQM	Relational quantum mechanics
RQD	Relational quantum dynamics
S(ρ∥σ)	Umegaki relative entropy (quantum KL; strictly convex in first argument)
$D_{\mathrm{KL}}\left(p∥q\right)$	Classical Kullback–Leibler divergence
$D_{\mathrm{JS}}^{Q}$	Quantum Jensen–Shannon divergence (symmetric; $\sqrt{D_{\mathrm{JS}}^{Q}}$ is a metric)
S(C)	Classical state space for context *C* (states diagonal in $\mathrm{alg}\{P_{i}\}$)
$E_{C}\left(\rho \right)$	Conditional expectation (dephasing) of ρ onto context *C*
$\Pi_{C}$	Conditional expectation map onto algebra *C* (pinching operator)
Φ(ρ)	Contextual divergence (min. weighted KL to NC; =0 iff noncontextual)
$\mu_{C}$	Positive weight for context *C* in the sum defining Φ ($\sum_{C}\mu_{C}=1$)
$p_{C}\left(\rho \right),\;g_{C}$	Born probabilities $p_{C}\left(i;\rho \right)=\mathrm{Tr}\left(\rho P_{i}\right)$ ; model marginals $g_{C}$ in context *C*
NC	Noncontextual polytope (empirical models admitting a global joint/section)
M	Measurement cover (family of contexts C⊂X)
$A_{C}$	Marginalization map from global *g* to $g_{C}$ (so $g_{C}=A_{C}g$)
$O_{EF}$	Overlap algebra generated by POVMs *E* and *F*
$M_{E}$	Classical measurement channel of POVM *E* (outputs $\mathrm{Tr}(\rho E_{i})$ on basis |i〉)

## Appendix A. Degenerate & POVM Contexts Survive Naimark Dilation

### Appendix A.1. Preliminaries and Notation

Let $E={\left\{E_{i}\right\}}_{i=1}^{m}$ be a POVM on a finite–dimensional Hilbert space *H* with $\sum_{i}E_{i}=1_{H}$. By Naimark’s theorem there exist an ancilla *K*, an isometry $V:H\;\to \;\hat{H}:=H⊗K$, and a commuting family of orthogonal projections ${\hat{P}}_{i}$ such that $E_{i}=V^{†}{\hat{P}}_{i}V$ [51]. Denote by $\hat{C}=\mathrm{alg}\left\{{\hat{P}}_{i}\right\}\subset B\left(\hat{H}\right)$ the resulting MASA and by

(A1) $$\Pi_{\hat{C}}\left(X\right)\;=\;\sum_{i}{\hat{P}}_{i}X{\hat{P}}_{i}$$

the (trace-preserving) conditional expectation (a.k.a., pinching) onto $\hat{C}$ [26,52]. Define the classical measurement channel

(A2) $$M_{E}\left(\rho \right)\;=\;\overset{m}{\underset{i=1}{⨁}}\mathrm{Tr}\left(\rho E_{i}\right)\;|i\rangle \;\langle i|,$$

whose adjoint is

(A3) $$M_{E}^{†}\;\left(\mathrm{diag}(x_{1},\ldots ,x_{m})\right)\;=\;\sum_{i}x_{i}E_{i}.$$

We use the Umegaki relative entropy S(τ∥ρ)=Tr[τ(logτ−logρ)], which is invariant under isometries, monotone under CPTP maps, and strictly convex in its *first* argument [25,53].

### Appendix A.2. KL Projection with Fixed POVM Statistics

#### Appendix A.2.1. Problem

Given ρ∈D(H), minimize the change to ρ measured by relative entropy while preserving the observed POVM statistics:

(A4) $$\min_{\tau \in D(H)}\;S\left(\tau ∥\rho \right)\;s.t.\;M_{E}\left(\tau \right)=M_{E}\left(\rho \right)\;\;⟺\;\;\mathrm{Tr}\left(\tau E_{i}\right)=\mathrm{Tr}\left(\rho E_{i}\right)\;\;\forall i.$$

**Theorem** **A1** (Minimum-change state under fixed POVM outcomes)**.** *The unique solution of Equation (A4) has the* exponential-family *form*

(A5) $$\tau^{★}\;=\;\frac{\exp\;\left(\log\rho -\sum_{i=1}^{m}\lambda_{i}E_{i}\right)}{\mathrm{Tr}\;\exp\;\left(\log\rho -\sum_{i=1}^{m}\lambda_{i}E_{i}\right)},\;\mathrm{with}\;\;\mathrm{Tr}\left(\tau^{★}E_{i}\right)=\mathrm{Tr}\left(\rho E_{i}\right)\;\;\forall i,$$

*for a (generally unique) vector of Lagrange multipliers ${\left(\lambda_{i}\right)}_{i}$.* Special cases. *(i) If $E_{i}=P_{i}$ are orthogonal projectors (a PVM), then $\tau^{★}=\sum_{i}\mathrm{Tr}\left(\rho P_{i}\right)\;P_{i}$ (conditional expectation onto the MASA). (ii) If all $E_{i}$ commute, $\tau^{★}$ belongs to the abelian algebra $\mathrm{alg}\{E_{i}\}$ and Equation (A5) reduces to a classical exponential family.*

**Proof sketch.** Form the Lagrangian $L\left(\tau ,\lambda \right)=S\left(\tau ∥\rho \right)+\sum_{i}\lambda_{i}\left(\mathrm{Tr}\left(\tau E_{i}\right)-\mathrm{Tr}\left(\rho E_{i}\right)\right)$. Stationarity on the manifold of density operators yields the exponential form Equation (A5); strict convexity of S(·∥ρ) in its first argument gives uniqueness on the common support [27,54]. The PVM case follows because the constraints commute and the minimizer collapses to the conditional expectation (pinching) [26]. □

#### Appendix A.2.2. Remark (Instruments vs. Variational Projection)

The completely positive map

(A6) $$L_{E}\left(\rho \right)\;=\;\sum_{i}\sqrt{E_{i}}\;\rho \;\sqrt{E_{i}}$$

is the post-measurement state of the canonical Lüders instrument for *E*. It generally does not solve Equation (A4) (nor preserve the *E*-statistics except in special cases); it coincides with $\tau^{★}$ only for PVMs (and certain commutative situations). See the SIC example in Appendix A.6 below.

### Appendix A.3. Quantum Jeffrey Updates Between Contexts

Let $E=\{E_{i}\}$ and $F=\{F_{j}\}$ be POVMs. Write the overlap algebra

(A7) $$O_{EF}\;=\;\mathrm{alg}{\left\{E_{i},F_{j}\right\}}^{\prime \prime }.$$

There are two natural (and distinct) variational updates:

(A) Preserve all expectations on the overlap algebra. Minimize S(τ∥ρ) subject to Tr(τX)=Tr(ρX) for all *X* in a generating set of $O_{EF}$. Then
  (A8) $$\tau^{★}\;=\;\frac{\exp(\log\rho -\Lambda )}{\mathrm{Tr}\;\exp(\log\rho -\Lambda )},\;\Lambda \in O_{EF}\;\;\mathrm{chosen}\;\mathrm{to}\;\mathrm{match}\;\mathrm{the}\;\mathrm{constraints}.$$
  when $O_{EF}$ is abelian (e.g., PVMs), $\tau^{★}$ equals the conditional expectation (pinching) of ρ onto $O_{EF}$ [26].
(B) Preserve the *F*–POVM outcome distribution. Minimize S(τ∥ρ) subject to $\mathrm{Tr}\left(\tau F_{j}\right)=\mathrm{Tr}\left(\rho F_{j}\right)$ for all *j*. The solution is the exponential family Equation (A5) with $\left\{E_{i}\right\}$ replaced by $\left\{F_{j}\right\}$.

In either case, Naimark dilations can be used to realize the constraints with commuting projectors, but the minimizer is still of exponential form; it is not generally the Lüders channel $\sum_{j}\sqrt{F_{j}}\rho \sqrt{F_{j}}$ unless *F* is a PVM. See also [55,56].

### Appendix A.4. Global Contextuality Divergence Is Naimark-Stable

For a POVM cover ${\left\{E^{\alpha }\right\}}_{\alpha \in C}$ define (as in the main text)

(A9) $$\Phi \left(\rho \right)\;=\;\min_{g\in \mathrm{NC}}\;\sum_{\alpha }\mu_{\alpha }\;D_{\mathrm{KL}}\;\left(p_{\alpha }\left(\rho \right)\;∥\;g_{\alpha }\right),$$

where $p_{\alpha }\left(\rho \right)$ are the Born probabilities for context α. If *V* is any Naimark isometry dilating all contexts to projective measurements $\left\{{\hat{P}}^{\alpha }\right\}$, then

(A10) $$\Phi_{E^{\alpha }}\left(\rho \right)\;=\;\Phi_{{\hat{P}}^{\alpha }}\;\left(V\rho V^{†}\right),$$

because Born probabilities are preserved by dilation ($\mathrm{Tr}\left(\rho E_{i}^{\alpha }\right)=\mathrm{Tr}\left(V\rho V^{†}{\hat{P}}_{i}^{\alpha }\right)$) and Φ depends only on those probabilities. Hence all results stated for PVM covers apply verbatim to POVM covers.

### Appendix A.5. Degenerate Projectors

If a projective context contains degenerate spectral projectors $P_{k}$ (rank >1), the conditional expectation is the block pinching

(A11) $$E_{P}\left(\rho \right)\;=\;\sum_{k}P_{k}\rho P_{k},$$

which is basis-independent within each block and remains the unique minimizer of S(τ∥ρ) over the block-diagonal algebra (strict convexity argument unchanged) [57,58].

### Appendix A.6. Illustrative Toy Example: Qubit Tetrahedral SIC

For the symmetric-informationally-complete POVM $E={\left\{\frac{1}{4}\left(1+{\vec{n}}_{i}\;\cdot \;\vec{\sigma }\right)\right\}}_{i=1}^{4}$ and a qubit state $\rho =\frac{1}{2}\left(1+\vec{r}\;\cdot \;\vec{\sigma }\right)$, the Lüders channel Equation (A6) gives

(A12) $$L_{E}\left(\rho \right)\;=\;\sum_{i=1}^{4}\frac{1}{4}\left(1+{\vec{n}}_{i}\;\cdot \;\vec{r}\;\right)\;\frac{1+{\vec{n}}_{i}\;\cdot \;\vec{\sigma }}{2}\;=\;\frac{1}{2}\left(1+\frac{1}{3}\;\vec{r}\;\cdot \;\vec{\sigma }\right),$$

i.e., a 1/3 shrink of the Bloch vector [59]. This does not preserve the SIC outcome probabilities unless ρ is maximally mixed, so $L_{E}\left(\rho \right)$ is not the minimizer of Equation (A4). The true minimizer is the exponential-family state Equation (A5) with multipliers $\left(\lambda_{i}\right)$ determined by the four linear constraints $\mathrm{Tr}\left(\tau^{★}E_{i}\right)=\mathrm{Tr}\left(\rho E_{i}\right)$.

## Appendix B. Rigorous Variational Proof (Finite-Context Setting)

This appendix replaces the informal argument in Section 3 with a fully rigorous derivation that: (i) formulates the optimization over the noncontextual feasible set; (ii) establishes existence of a minimizer using lower-semicontinuity on a compact domain (no hand-waving); (iii) handles zero-probability coordinates via complete KKT conditions; (iv) proves uniqueness of the optimal marginals by strict convexity; and (v) identifies when the unique optimal marginals coincide with the Born distributions, and how informational completeness then yields the underlying state.

### Appendix B.1. Setting and Notation

- Hilbert space: $H≅C^{d}$; density matrix ρ∈D(H).
- Context cover: $M=\{C_{1},\ldots ,C_{m}\}$, each $C_{j}=\left\{P_{j,1},\ldots ,P_{j,d}\right\}$ a rank-1 PVM with $\sum_{i}P_{j,i}=1$.
- Born (context-wise) distributions: $p_{C_{j}}\left(i\right):=\mathrm{Tr}\left(\rho P_{j,i}\right)$.
- Deterministic global assignments: let Ω be the (finite) set of functions ω that assign to each context $C_{j}$ precisely one outcome index ω(j)∈{1,…,d} and are context-consistent on overlaps (if $P_{j,i}=P_{k,\ell }$, then ω(j)=i iff ω(k)=ℓ). (This is the usual “global section” set in the sheaf model; Ω may be empty in strongly contextual scenarios. In that case, one can enlarge the cover (e.g., add symmetric white-noise coarse-graining) to restore feasibility; here we assume Ω≠⌀ so the noncontextual set is nonempty.)
- Noncontextual polytope (global variable): $\Delta_{\Omega }:=\left\{g\in R_{\geq 0}^{\Omega }:\sum_{\omega \in \Omega }g_{\omega }=1\right\}$.
- Context-wise marginals of *g*: for each *j* and *i*,
  $$\left(A_{j}g\right)\left(i\right)\;:=\;\sum_{\omega \in \Omega :\;\omega (j)=i}g_{\omega },\;\mathrm{so}\;g_{C_{j}}:=A_{j}g\in \Delta_{C_{j}}.$$
  Here $A_{j}$ is the marginalization matrix from $R^{\Omega }$ to $R^{d}$.
- Weights: $\mu_{j}>0$ with $\sum_{j}\mu_{j}=1$.
- Objective (global “glue” cost):
  (A13) $$F\left(g\right)\;:=\;\sum_{j=1}^{m}\mu_{j}\;D_{\mathrm{KL}}\left(p_{C_{j}}\;∥\;A_{j}g\right)\;=\;\sum_{j=1}^{m}\sum_{i=1}^{d}\mu_{j}\;p_{C_{j}}\left(i\right)\;\ln\frac{p_{C_{j}}\left(i\right)}{\left(A_{j}g\right)\left(i\right)}.$$

We minimize *F* over the noncontextual set $\Delta_{\Omega }$. This enforces both per-context normalization and global consistency (all $g_{C_{j}}$ are marginals of a single *g*).

### Appendix B.2. Existence of a Minimizer

We view *F* as an extended-value function by the usual KL convention: $p\log\frac{p}{y}$ is +∞ when p>0 and y=0, and 0 when p=0 and y≥0.

**Lemma** **A1** (Lower-semicontinuity and coercivity on the boundary)**.** *For each j, the map $g\mapsto D_{\mathrm{KL}}\left(p_{C_{j}}∥A_{j}g\right)$ is lower-semicontinuous on $\Delta_{\Omega }$; moreover, it diverges to +∞ along any sequence in $\Delta_{\Omega }$ for which $\left(A_{j}g\right)\left(i\right)↓0$ with $p_{C_{j}}\left(i\right)>0$.*

**Proof.** The map $g\mapsto A_{j}g$ is linear and continuous. On [0,1], y↦plog(p/y) is lower-semicontinuous for each fixed p≥0, with the stated extended value; composition with a continuous map preserves lower semicontinuity; finite nonnegative sums preserve it. The divergence claim is immediate from plog(p/y)→+∞ as y↓0 for p>0. □

**Proposition** **A1** (Existence of an optimal noncontextual model)**.** *F attains its minimum on $\Delta_{\Omega }$.*

**Proof.** $\Delta_{\Omega }$ is a nonempty compact simplex. By Lemma A1, *F* is lower-semicontinuous on $\Delta_{\Omega }$ and not identically +∞ (e.g., take *g* uniform on Ω, which yields strictly positive marginals). Hence *F* achieves its infimum by the Weierstrass theorem; see, e.g., Theorem 1.9 in [60] and Section 2.7 in [54]. □

### Appendix B.3. Uniqueness of Optimal Marginals via Strict Convexity

**Lemma** **A2** (Strict convexity in the marginals)**.** *For fixed $p\in \Delta_{C_{j}}$, the function $y\mapsto D_{\mathrm{KL}}\left(p∥y\right)$ is strictly convex on $\left\{y\in \Delta_{C_{j}}:y_{i}>0\;\mathrm{whenever}\;p_{i}>0\right\}$. Consequently,*

$$y\;:=\;{\left(y^{\left(j\right)}\right)}_{j=1}^{m}\;⟼\;\sum_{j=1}^{m}\mu_{j}\;D_{\mathrm{KL}}\left(p_{C_{j}}\;∥\;y^{\left(j\right)}\right)$$

*is strictly convex on the Cartesian product of those interiors.*

**Proof.** The Hessian of $y\mapsto -\sum_{i}p_{i}\log y_{i}$ is $\mathrm{diag}(p_{i}/y_{i}^{2})$, positive definite on the stated domain. □

**Proposition** **A2** (Uniqueness of optimal marginals)**.** *Let $g^{★}$ minimize F on $\Delta_{\Omega }$. Then the* vector of marginals *${\left(A_{j}g^{★}\right)}_{j=1}^{m}$ is unique. If two minimizers $g^{\left(1\right)},g^{\left(2\right)}$ exist, they have identical marginals $A_{j}g^{\left(1\right)}=A_{j}g^{\left(2\right)}$ for all j (they may differ along the affine fiber $\left\{g:A_{j}g=A_{j}g^{★}\;\forall j\right\}$).*

**Proof.** *F* depends on *g* only through $y:={\left(A_{j}g\right)}_{j}$. The image set $Y:=\{{\left(A_{j}g\right)}_{j}:g\in \Delta_{\Omega }\}$ is convex and compact. By Lemma A2 the objective in y is strictly convex on the relevant domain, hence admits a unique minimizer $y^{★}\in Y$. Any two *g* that minimize *F* must map to the same $y^{★}$. □

### Appendix B.4. KKT Characterization with Zeros on the Support

Write the Lagrangian with a single global variable $g\in \Delta_{\Omega }$:

$$L\left(g,\lambda ,\nu \right)=\sum_{j=1}^{m}\mu_{j}\sum_{i=1}^{d}p_{C_{j}}\left(i\right)\;\ln\frac{p_{C_{j}}\left(i\right)}{\left(A_{j}g\right)\left(i\right)}\;+\;\lambda \left(\sum_{\omega \in \Omega }g_{\omega }-1\right)\;-\;\sum_{\omega \in \Omega }\nu_{\omega }g_{\omega },$$

where λ∈R enforces $1^{⊤}g=1$ and $\nu_{\omega }\geq 0$ enforce $g_{\omega }\geq 0$.

Stationarity

For any optimal $g^{★}$ in the (relative) interior of its support,

(A14) $$\nabla_{g}L\left(g^{★},\lambda ,\nu \right)=\sum_{j=1}^{m}\mu_{j}\;A_{j}^{⊤}\left(-\;\frac{p_{C_{j}}}{A_{j}g^{★}}\right)\;+\;\lambda \;1\;-\;\nu \;=\;0,$$

with the fraction taken componentwise. Coordinates *i* with $p_{C_{j}}\left(i\right)=0$ simply do not contribute to the gradient.

#### Appendix B.4.1. Complementary Slackness and Feasibility

(A15) $$\nu_{\omega }\;g_{\omega }^{★}=0\;\forall \omega ,\;\nu_{\omega }\geq 0,\;g_{\omega }^{★}\geq 0,\;\sum_{\omega }g_{\omega }^{★}=1.$$

Moreover, $F(g^{★})<\infty$ forces $\left(A_{j}g^{★}\right)\left(i\right)=0$ whenever $p_{C_{j}}\left(i\right)=0)$ is allowed, but $\left(A_{j}g^{★}\right)\left(i\right)>0$ is *required* whenever $p_{C_{j}}\left(i\right)>0)$.

#### Appendix B.4.2. Interpretation (Csiszár I-Projection)

Equation (A14) is the optimality condition that the image ${\left(A_{j}g^{★}\right)}_{j}$ be the (weighted) Csiszár *I*-projection of the Born bundle ${\left(p_{C_{j}}\right)}_{j}$ onto the convex set $Y=\{{\left(A_{j}g\right)}_{j}:\;g\in \Delta_{\Omega }\}$; see [27,54]. When Ω separates images (e.g., columns of the stacked matrix $A:={\left[A_{1}^{⊤}\;\ldots \;A_{m}^{⊤}\right]}^{⊤}$ are affinely independent over the face touched by $g^{★}$), the minimizer $g^{★}$ itself is unique; otherwise, the optimal fiber $\left\{g:A_{j}g=A_{j}g^{★}\;\forall j\right\}$ is a (possibly higher-dimensional) face. A canonical selector is the maximum-entropy point on that face.

### Appendix B.5. When Does the Minimizer Equal the Born Distributions?

**Proposition** **A3** (Characterization of equality)**.** *Let $g^{★}$ minimize F on $\Delta_{\Omega }$, and let $p\left(\rho \right):={\left(p_{C_{j}}\right)}_{j}$. Then the following are equivalent:*

(a) *$A_{j}g^{★}=p_{C_{j}}$ for all j (the optimal marginals equal the Born distributions in every context).*
(b) *p(ρ)∈Y, i.e., the Born bundle is* noncontextual *(admits a global joint $g\in \Delta_{\Omega }$).*

*In this case the minimum value is $F(g^{★})=0$ and any optimizer is supported on the affine fiber $\left\{g:A_{j}g=p_{C_{j}}\;\forall j\right\}$.*

**Proof.** (b)⇒(a): If p(ρ)∈Y, choose *g* with $A_{j}g=p_{C_{j}}$; then F(g)=0 is the global minimum, and by uniqueness of optimal marginals (Proposition A2) any optimizer must have $A_{j}g^{★}=p_{C_{j}}$. (a)⇒(b): Trivial, since ${\left(A_{j}g^{★}\right)}_{j}=p\left(\rho \right)\in Y$ by feasibility of $g^{★}$. □

When ρ is contextual for the chosen cover (so p(ρ)∉Y), Propositions A2 and A3 imply that $A_{j}g^{★}$ deviates from $p_{C_{j}}$ on at least one context and $F(g^{★})>0$.

### Appendix B.6. Informational Completeness and Reconstruction of ρ

**Definition** **A1** (Informational completeness (IC))**.** *The cover M is informationally complete if $\mathrm{span}\left\{P_{j,i}:\;1\leq j\leq m,\;1\leq i\leq d\right\}=B\left(H\right).$ Equivalently, the linear map $\rho \mapsto {\left(\mathrm{Tr}(\rho P_{j,i})\right)}_{j,i}$ is injective.*

**Corollary** **A1** (Reconstruction under IC)**.** *If M is IC and p(ρ)∈Y (noncontextual case), then any minimizer satisfies $A_{j}g^{★}=p_{C_{j}}$ for all j, and these equalities determine ρ uniquely via linear inversion on the IC frame $\left\{P_{j,i}\right\}$. In particular, the variational program picks out the* unique *quantum state consistent with the optimal marginals.*

**Remark** **A1.**
*(i) The results above remain valid for degenerate PVMs if one replaces $P_{j,i}$ by the normalized block states $\pi_{j,i}:=P_{j,i}/\mathrm{rank}P_{j,i}$ in the definitions of $A_{j}$ and $p_{C_{j}}$; the proofs are unchanged. (ii) For POVMs, the global-variable formulation still applies once $A_{j}$ is defined by the corresponding classical post-processing; the uniqueness of optimal marginals remains a consequence of strict convexity of $D_{\mathrm{KL}}$ in its second argument.*
